# Loneliness, Depression, and Genetics in the Elderly: Prognostic Factors of a Worse Health Condition?

**DOI:** 10.3390/ijerph192315456

**Published:** 2022-11-22

**Authors:** María Luisa Delgado-Losada, Jaime Bouhaben, Eduardo Arroyo-Pardo, Aránzazu Aparicio, Ana María López-Parra

**Affiliations:** 1 Experimental Psychology, Cognitive Processes and Speech Therapy Department, Faculty of Psychology, Complutense University of Madrid, 28223 Pozuelo de Alarcón, Spain; 2 Group of Neurosciences: Psychoneuroendocrinology, Neuroimaging and Molecular Genetics in Neuropsychiatric Diseases, Instituto de Investigación Sanitaria San Carlos (IdISSC), Hospital Clínico de Madrid, 28040 Madrid, Spain; 3 UCM Research Group: VALORNUT-920030, Department of Nutrition and Food Science, Faculty of Pharmacy, Complutense University of Madrid, Plaza Ramón y Cajal s/n, 28040 Madrid, Spain; 4 Laboratory of Forensic and Population Genetics, Legal Medicine, Psychiatry and Pathology Department, Medicine School, Complutense University of Madrid, Plaza Ramón y Cajal s/n, 28040 Madrid, Spain; 5 Group of Forensic Sciences: Forensic Genetics and Toxicology, Instituto de Investigación Sanitaria San Carlos (IdISSC), Hospital Clínico de Madrid, 28040 Madrid, Spain; 6 Department of Nutrition and Food Science, Faculty of Pharmacy, Complutense University of Madrid, Plaza Ramón y Cajal s/n, 28040 Madrid, Spain

**Keywords:** loneliness, depression, health, ELES study, telomeres, SNPs, BDNF, elderly

## Abstract

Loneliness is considered a prognostic factor for poorer health status in the elderly. It is proposed to analyze the role of loneliness in health status in terms of various factors. A total of 1747 individuals from the pilot survey of the Aging in Spain Longitudinal Study (ELES-PS) were reviewed. ELES is a cross-sectional study for collecting health variables, food habits, socioeconomic data, and cognitive and functional capacities, which was carried out on a Spanish representative sample of noninstitutionalized persons of 50 years of age or older. Moreover, since telomere shortening is associated with cellular senescence, 35 telomere-related SNPs and cognitive impairments were analyzed. The results characterize the “solos” as males of 50–60 years, who were overweight and had lower levels of hemoglobin and neutrophils. There is also an association between five SNPs related to telomere length and BDNF. A group of people with loneliness and depression was identified with poorer health and cognitive status, poorer perception of their quality of life, poorer quality of sleep, and lower physical activity. Therefore, it follows that telomeres and BDNF play a role as intermediaries between loneliness and depression and their relationship with a worse state of health.

## 1. Introduction

Loneliness is a universal human experience [1] and is an important worldwide public health concern affecting people of all cultures and ages. It is considered to be one of the concerns in our society due to the fact that it has very negative consequences on people’s quality of life [2,3]. Tackling loneliness is a key part of the successful global response to the third goal of the UNSDG 2030 Agenda, namely, to ensure healthy lives and promote well-being at all ages [4].

Loneliness is a complex experience and difficult to define. It has been defined by Peplau and Perlman (1982) [5] and also by Weiss (1973) [6] as an unpleasant feeling resulting from an individual’s perceived or actual deficiency in the desired quality of their social relationships. For this reason, loneliness is often distinguished from mere being alone. This is why loneliness can generate feelings of boredom, exclusion, and marginality [7,8,9,10].

### 1.1. Loneliness and Negative Health Outcomes

Holt-Lunstad and Smith (2016) [11] postulate the existence of three theoretical pathways that explain the factors underlying the association between feelings of loneliness and negative health outcomes. The biological pathway is related to the biological and physiological processes associated with the prevalence of a wide range of diseases. The psychological pathway considers a set of factors such as lack of social support or depression that explain part of the association between loneliness and negative health outcomes. The behavioral pathway associates factors related to lifestyle, eating habits, alcohol consumption, and sleep patterns as factors that explain part of these associations [12,13].

Several examples can be produced. Loneliness is a risk factor for cardiovascular diseases [14,15] since it increases vascular resistance [16], elevates blood pressure and the possibility of recurrent strokes [16,17], and the risk of obesity [18,19]. It is also associated with an increased hypothalamic pituitary adrenocortical activity [20], a diminished immunity [21,22], an underexpression of genes bearing anti-inflammatory glucocorticoid response elements and an upregulation of proinflammatory gene transcripts [23,24], abnormal ratios of circulating white blood cells (e.g., neutrophils, lymphocytes, and monocytes [25]), and premature mortality (e.g., [26,27]).

The presence of recurrent feelings of loneliness affects mental health and psychological functioning [28] and has been identified as a risk factor for the onset of depressive symptoms [29,30,31], also closely aligned with, and possibly prodromal to, depression [32]. In addition, loneliness has been identified as a risk factor for low life satisfaction [33], poor sleep patterns—which, in turn, affect the body’s restorative processes [7]—aggressive behaviors, social anxiety, and impulsivity [34,35].

In relation to cognitive health, in the systematic review of Boss, Kang and Branson (2016) [36], associations of loneliness and cognitive function in the aging population are reviewed to find that loneliness is negatively associated with cognitive function. The main findings were significant and showed negative correlations of loneliness with global cognitive function or general cognitive ability [37,38,39,40,41,42], intelligence quotient (IQ) [41,43], processing speed [41,43,44], immediate recall [44,45], and delayed recall [45,46].

### 1.2. Loneliness and Sociodemographic Factors

There are some sociodemographic characteristics that seem to be related to loneliness, among which sex, marital status, age, and place of residence seem to have greater evidence. Regarding sex, Holwerda et al. (2012) [40] claim that the prevalence of feelings of loneliness in women is more than twice as high as in men, due to the greater longevity of women together with their greater exposure to the loss of the spouse or of other people from the same generation.

It has been consistently shown that feelings of loneliness are more prevalent among the single or the widowed [47,48], and concerning age, loneliness followed a bimodal U-shaped distribution [48,49,50]. In fact, it is more prevalent in young adults (18–25 years) and older adults (>65 years old), as Martín-Roncero (2020) [50] has confirmed in the Spanish population. However, Luhmann and Hawkley (2016) [51] observed a more complex scenario, with two peaks of loneliness at 30 and 60, and two decreases at 40 and 75, finding the lowest levels at 75.

### 1.3. Loneliness and Genetic Factors

With regard to the genetic basis of loneliness, Boomsma estimated a higher percentage of the heritability for loneliness (48%) based on adult twin data [52]. More recently, a GWAS found that a heritability of loneliness in the range of 14–27% in 12,454 Americans who were >50 years of age [53]. Moreover, there is evidence that certain candidate genes are associated with loneliness: Day et al. (2018) identified 15 genomic loci in a GWAS in the UK Biobank study (487,647 individuals) [54].

Several studies sought to identify the contribution of specific genes to feelings of loneliness, focusing on genes related to neurotransmitters such as dopamine, serotonin, or other attachment-related cellular systems such as oxytocin [55,56,57]. Another author argued that there is a common genetic basis with various mental illnesses such as schizophrenia, major depression, bipolar depression, and loneliness [53]. Although depression has been considered as another factor in some of these studies, to our knowledge, statistical analyses have not been carried out considering loneliness and depression together.

The telomere shortening is related to cell age. The older people are, the shorter their telomeres are. The maintenance of telomere length is key since excessive shortening would lead to the appearance of symptoms associated with premature aging, and aberrant activity could favor the immortality of malignant cells. In recent years, there has been a number of publications on the search for mutations in the TER (RNA sequence of telomerase) sequence and in the TERT (telomerase reverse transcriptase) gene in various pathologies such as Zinsser–Cole–Engmann syndrome, also called Dyskeratosis Congenita [58]; aplastic anemia [59]; different types of cancer such as glioblastoma [60], melanoma [61], thyroid carcinoma [62], and breast cancer [63], among others; idiopathic pulmonary fibrosis [64]; and atherosclerosis [65] and more. Carroll et al. (2013) [66] observed that elderly people (65–84 years), both men and women, with less social support had shorter telomeres. According to these data, loneliness could favor the greater shortening of telomeres. The effect that certain polymorphisms related to telomere shortening and loneliness may have in individuals with depression is not yet known.

Therefore, the present work aims at analyzing the role that loneliness plays in the state of health of single people, in terms of different genetic, cognitive, biochemical, and lifestyle factors.

First of all, in particular, it is specifically proposed to research the association between SNPs related to telomerase activity and loneliness. The second specific objective is to establish the profile (genetic, biochemical, cognitive, activity, etc.) of individuals classified as solos. The third specific objective focuses on defining the effect of loneliness on physical and psychological health.

## 2. Materials and Methods

### 2.1. Study Design

The pilot survey of the Aging in Spain Longitudinal Study, Pilot Survey (ELES-PS) is a cross-sectional study conducted in Spain, designed to collect health variables, food habits, socioeconomic data, and cognitive and functional capacities to analyze the aging process. The ELES study was carried out on a Spanish representative sample of noninstitutionalized persons of 50 years of age or older. Details of the study were published elsewhere [67,68,69,70].

With regard to ethical aspects, the ELES was approved by the Bioethics Subcommittee of the Spanish National Research Council, and the protocol of this study was approved by the Ethics Committee of Hospital Clínico San Carlos (ref. 17/125-E). Prior to the surveys, an informed consent form was granted and signed by each respondent, and anonymity was assured.

### 2.2. Participants

The target population was people over 50 years of age, noninstitutionalized and resident in the national territory. Data collection was performed in two parts: The first part consisted of a computer-assisted telephone interview (CATI) in which personal data (sex and age) were collected. The second part of the study consisted of a nurse visit, where blood samples and anthropometric measurements were taken and the Mini Mental-State Examination [71] and a computer-assisted personal interview (CAPI) were performed.

The final sample size was 1747 telephone interviews with people who initially participated in the study. From this initial sample of 1747 interviewees, 216 cases refused to continue in the study after the telephone interview, not accepting the visit of nursing staff, which implied a final sample of 1531 individuals. Of the 1531 participants, 1185 people were assessed for global cognitive status, and 1072 people completed the assessment of cognitive function. 

Two databases were built. The first one had a size of 414 individuals in which only those who underwent genetic analysis were included, and the second one, which corresponds to the total database, comprised 999 individuals. Only 999 participants had data on levels of loneliness and depression.

The samples from which the DNA was obtained had previously been stored in the Basque Biobank created by the Foundation for Health Innovation and Research (BIOEF, Fundación Vasca de Innovación e Investigación Sanitaria) (https://www.biobancovasco.org). All the analyses were previously approved by the ethical and scientific committees of the Basque Biobank.

### 2.3. Measurements

The information for the ELES-PS was collected in four phases: (i) telephone questionnaire; (ii) visit by nurses (blood and saliva samples, administration of a cognitive deterioration screening, measurement of skin folds, and recording of medicines taken by the individual); (iii) CAPI questionnaire (computer-assisted personal interviewing) conducted by trained interviewers and taking of anthropometric measurements and performing tests; and (iv) self-administered questionnaire (http://www.proyectoeles.es accessed on 1 March 2022).

#### 2.3.1. Assessments of Sociodemographic Variables and Health Conditions

Baseline sociodemographic information included age, sex, marital status, education level, and habitat.

The diseases were recorded by means of a self-report, including hypertension, diabetes, cardiac diseases, cancer, lung disease, heart disease, musculoskeletal diseases, diabetes, stomach or duodenal ulcer, kidney disease, depression, anxiety, embolism or stroke, cancer, high cholesterol, Parkinson’s disease, mobility difficulties, allergies, and urinary tract problems.

This included a question about the area of the body in which they felt pain that may have made it difficult for them to carry out their usual work or leisure activities, and other factors such as sleep quality, height and weight, and biochemical parameters such as hemoglobin levels or cholesterol.

The ELES-PS used the Yale Physical Activity Survey (YPAS) [72] as a measure of physical activity. The satisfaction with life was evaluated through a series of questions related to their standard of living, health status, achievements in life, personal relationships, feeling of security, sense of belonging to the community, and spiritual life. The amount of sleep was assessed through the question “how many hours do you sleep on average per day?”, and the quality of sleep by asking “How often do you wake up rested in the morning?” and “How many times in the last week have you had difficulty falling asleep, woke up several times in your sleep, or woken up too soon?”

#### 2.3.2. Assessments of Loneliness and Depression

Feelings of loneliness were measured by the De Jong Gierveld Scale for Loneliness (DJGLS) [73], which was designed to measure overall, emotional, and social loneliness. The six-item version includes three negatively worded items and three positively worded items. A cutting score of 3 was used to distinguish not lonely from lonely people [74]. This allowed SOLTOT and SOLTOT2G to be defined. SOLTOT is a continuous variable with values from 0 to 6. SOLTOT2G is a dichotomous variable, where 0 means loneliness and 1 means no loneliness.

Depression was evaluated using the 10-item version of the Center for Epidemiological Studies Depression Scale (CES-D 10) [75]. A cutting score of 5 was used to distinguish not depression from depressed people [76]. Individuals with CESD greater than or equal to 5 are considered to have depression.

#### 2.3.3. Cognitive Assessments

The cognitive function was assessed through the following five performance tests: MMSE was used to measure global cognitive function of the participants, such as orientation, attention, memory, language, and visual–spatial skills [71]. To conduct this assessment, we employed: a word list of immediate and delayed verbal recall from the Auditory Verbal Learning Test as a measure of verbal memory (AVLT) [77]; Boston Naming Test as a measure of semantic memory [78]; a cancellation task as a measure of the processing speed of the information, adapted from the letter cancellation task of the ELSA study, which provides an index of processing speed; and a verbal fluency task, semantic and phonological, for assessed language, semantic memory, and executive function [79]. Participants were asked to name as many animals as possible and as many words beginning with the letter “s” as possible within a 60 s period.

#### 2.3.4. Genetic Analysis

DNA was extracted with SPEEDTOOLS DNA EXTRACTION KIT (Biotools) in 414 samples. All SNPs except rs7412 were genotyped by means of the Sequenom technique, using iPLEX chemistry. SNP genotyping services were provided by the Spanish “National Genotyping Centre” (CEGEN-USC, http://www.cegen.org) rs7412 was sequenced using normalized protocols from the lab [80].

#### 2.3.5. Selection of SNPs

A total of 28 SNPs were selected from the literature related to telomere shortening. In addition, 7 SNPs related to cognitive diseases such as Alzheimer’s have been added.

### 2.4. Statistical Analysis

For the achievement of the objectives, the analysis was carried out in 2 levels.

In the first level, 2 groups were defined: control group (SOLTOT2G = 0) (*n* = 129) and solos group (SOLTOT2G = 1) (*n* = 191) from a database of 414 individuals. Only 320 participants had information on loneliness. In these groups, it was assessed whether there were significant differences (*p* ≤ 0.05) between groups with the calculation of average, standard deviation, and F (test) for continuous variables and Pearson chi-squared test for ordinal and nominal variables. It was performed by using SPSS v28.

SNP allele frequencies assessment, genotype frequencies and Hardy–Weinberg equilibrium (HWE) were carried out using the R package (http://www.Rproject.org/).

Linear and logistic regression were calculated with SOLTOT using SNPSTAT, testing different models [81].

From the SNPs that were found to have an effect on total loneliness (SOLTOT), GRS was calculated (genetic risk score) [82], which was used in multivariate analyses such as regression and decision trees.

The association between genotype and phenotype variables was studied by decision trees using the Modeler version 18.2.2 statistical package. This methodology was used in order to determine the most discriminant variables among the different degrees of loneliness. Therefore, a decision tree was tested for loneliness variables (SOLTOT2G). The decision trees were built by means of a nonparametric method that clusters the observations according to a factor or predictor that better explains the differences in the studied variables. Each generated subdivision is again partitioned according to the existence of new predictors with a significant effect; thus, the data are presented in a hierarchical manner [83]. This method extracts information by finding effects of the factors that are not homogeneous at all the dependent variable levels and by discovering specific interactions between variables or data mining. From all the different analyses available for this partitioning approach, those algorithms that apply nonbinary divisions are more convenient. In this study, the CHAID (chi-square automatic interaction detection) exhaustive method was selected in Modeler. The significant threshold was *p* < 0.05, the minimum size of a node that could be divided; it consisted of 50 individuals, and the minimum size of a child node consisted of 25 individuals. Finally, this method applied corrections due to multiple testing through the Bonferroni method.

Associations between these independent variables and loneliness were studied through ordinal logistic regression models, as dependent variables were ordinal (loneliness is measured through a 5-choices Likert scale). Three exploratory models were computed, one for each dependent variable: social loneliness, emotional loneliness, and total loneliness. The logistic function was chosen as the loss function. Predictors’ selection in the final models was performed by a forward stepwise method based on the models’ AIC. All these statistical analyses were performed with R software, version 3.5.2. Regarding significance testing, the alpha level was set to 0.05 (α = 0.05).

A second level was established for the achievement of the third objective, and the total sample was divided into 4 groups: control group (*n* = 339), depressed individuals group (*n* = 66), solos group (*n* = 444), and “solos and depressed individuals group” (*n* = 150) in the full database. These groups were defined according to SOLTOT2G and the CESD score. The control group would have a value of SOLTOT = 0 and CESD < 5. The depressed individuals group would have a value of SOLTOT = 0 and CESD ≥ 5. The solos group would have a value of SOLTOT = 1 and CESD < 5. The solos and depressed individuals group would have a value of SOLTOT = 1 and CESD ≥ 5. In these groups, it was assessed whether there were significant differences, assuming *p* ≥ 0.05, between the groups with respect to the mean parameters. It was performed by using SPSSv28. Linear and logistic regression were calculated between the 4 groups and SNPS in the reduced database using SNSTAT again to test different models between these 4 groups. The sample sizes of the 4 groups in the reduced database were: control group (*n* = 120), depressed individuals group (*n* = 5), solos group (*n* = 153), and “solos and depressed individuals group” (*n* = 31). The corresponding decision trees were built.

## 3. Results

*First Level*

### 3.1. Descriptive Analysis

Table 1 shows the descriptive analyses of the reduced database. In the sample selected, it was found that there are significant differences with respect to sociodemographic variables in marital status when comparing the control group with the solos one. In this variable, 67% of widowers present loneliness. With regard to health status, only significant differences have been found in blood circulation problems and back pain, despite the high number of pathologies assessed in the questionnaires and the types of pain studied. Regarding cognitive parameters, significant differences are centered on the memory and verbal fluency of animals. Regarding the level of satisfaction, the solos group presented lower levels of satisfaction, which were statistically significant with respect to their life in general terms, state of health, personal relations, friendly relations, use of free time, and social support. Only in the biochemical parameters of MCHC (mean corpuscular hemoglobin concentration) and RDW (red cell distribution width) are opposite values observed; thus, the solos group presents lower values in MCHC and higher values in RDW with respect to controls. Finally, the quality of sleep is worse in the solos group.

**Figure 1:**
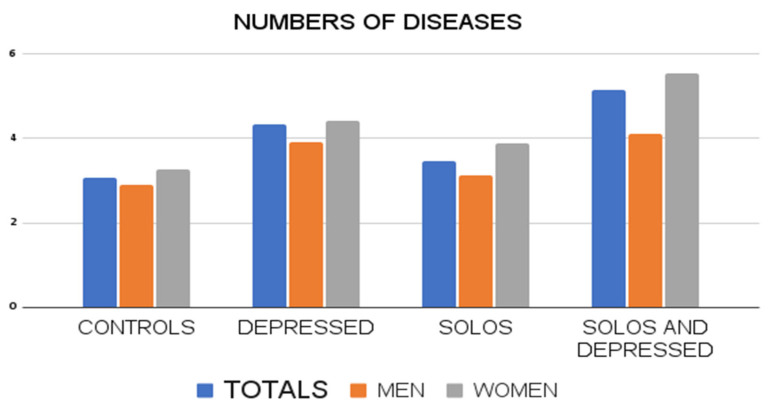
Graph of the number of total diseases in volunteers according to groupings (controls, depressed, solo, solo and depressed) and sex (total, men and women).

When considering only the number of diseases, it shows a greater number of diseases in the group of “solos and depressed individuals”, compared to healthy, solos, and depressed individuals group, with significant differences (Figure 1).

By differentiating by sex, similar results are observed, especially in the case of women, whose results are practically coincident with the comparisons in the total database, finding even more differences with respect to the “alone and depressed”. That is, the group of women with loneliness and depression have a worse state of health.

### 3.2. SNPS and Loneliness

Table 2 lists the allele and genotype frequencies as well as the *p*-values for the HWE test for the 36 studied SNPs. The 36 SNPs presented a minimum allele frequency (MAF) greater than 0.01. rs7412, rs3764650, rs2487999 presented a genotype with a frequency less than 0.01. Only rs429358 presented a HWE with a p lesser than 0.05. This SNP, with rs7412, permits genotyping APOE.

The logistic regression analysis results for all variables with SOLTOT are shown in Table 3. Associations have been found with six SNPs: rs2487999, rs391300, rs6265, rs12335203, rs2154110, and rs4902100. The association models are different in each case: log-additive (rs2487999 and rs12335203), dominant (rs391300), codominant (rs6265 and rs4902100), and overdominant (rs2154110). Within these SNPs, all but rs6265 directly or indirectly determine the length of telomeres. The GRS was calculated only in these six SNPS, considering the different inheritance models.

### 3.3. Multivariable Analysis 

#### 3.3.1. Logistic Regression

The results of the ordinal logistic regression analysis on the total loneliness variable (SOLTOT) are presented. Table 4 shows the coefficients of the predictor variables that make up the model with total loneliness. Regarding biological predictors, the effect of GRS stands out (b = 0.195, t = 4.545, *p* < 0.0001). Other biological or clinical variables in the model are hemoglobin (b = −0.233, t = −2.5, *p* = 0.012), RDW (b = 0.299, t = 2.708, *p* = 0.007), neutrophils (b = −0.032, t = −2.445, *p* = 0.015), sex (woman) (b = −1.093, t = −4.071, *p* < 0.0001), age (b = −0.819, t = −2.573, *p* = 0.01), height (b = 0.021, t = 2.56, *p* = 0.011), weight (b = −6.181, t = −4.993, *p* < 0.0001), and back pain (b = 0.145, t = 2.94, *p* = 0.003). A cognitive level highlights the effect of the general cognitive status (MMSE), categorized into MMSE = [27,30] versus MMSE <24 (b = 2.433, t = 2.24, *p* = 0.025), as well as in memory, three-word delay recall (b = −0.43, t = −2.539, *p* = 0.011), and in the processing speed measured with the cancellation task (b = −0.026, t = −2.981, *p* = 0.003). At the psychosocial level, there is a significant effect of the habitat (b = 0.694, t = 2.801, *p* = 0.005) and life satisfaction (b = −0.33, t = −4.323, *p* < 0.0001).

#### 3.3.2. Decision Trees

**Figure 2:**
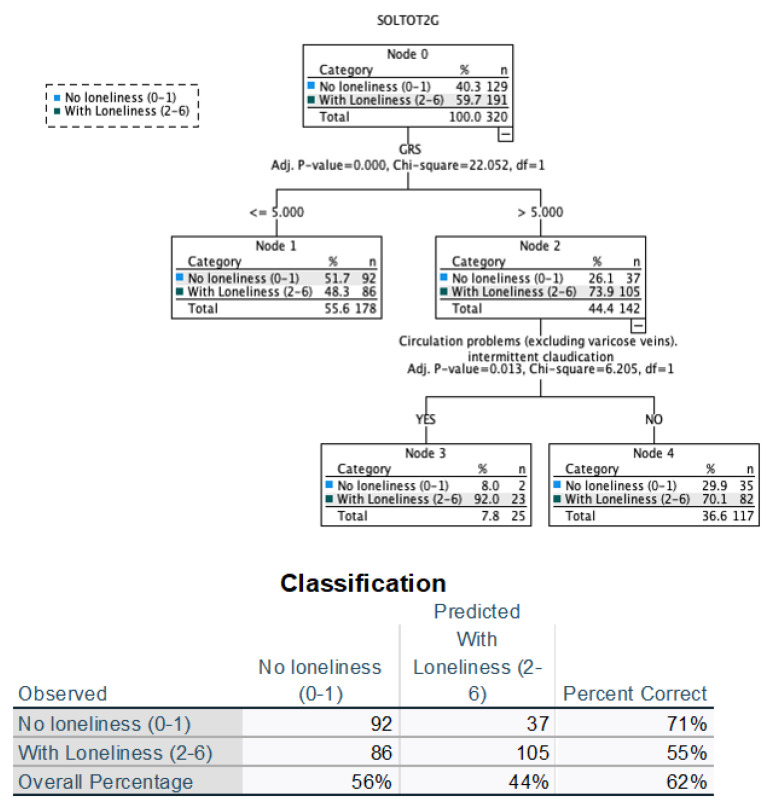
Decision tree and table of classification of objective variables related to health.

First, a decision tree was built considering only variables related to health status in an objective way: biochemical parameters, pathologies diagnosed. In the tree (Figure 2), it is observed that the first parameter that classifies between those with greater and lesser loneliness is the genetic risk. The second classification variable is circulation problems. It has a high sensitivity percentage (71.3%) and lower specificity (55%). By repeating the analysis including all the variables, the sensitivity of the tree drops to 36.4%, although the sensitivity increases (88.5%), and so this tree was discarded.

*Second level*

### 3.4. Descriptive Analysis

Supplementary Materials (Table S1) shows the descriptive analyses in relation to the total database for the four control groups, depressed individuals group, solos group, and “solos and depressed individuals group” (Table S1a–c is available in Supplementary Materials S1). The table only includes significant differences from the group of “lonely and depressed”. In all parameters related to health status, the “solos and depressed” group has higher values than the rest of the groups. When compared with the group of depressed individuals, they present significant differences in the assessment of health status, osteoporosis, and urinary tract problems. The number of pathologies, among which there are significant differences, is increased when compared with the solos group (osteoarthritis, arthritis, asthma, anxiety, and back pain). The same applies to biochemical parameters or satisfaction levels, with more significant comparisons observed for solos group and “solos and depressed individuals group”. The group of solos have values lower than the “solos and depressed individuals group” only regarding memory and do not present significant differences between them. When we repeat the analyses considering sex, in the case of men, there are hardly any significant differences between the group of depressed individuals and the group of “solos and depressed individuals” (Table S2a–c is available in Supplementary Materials S2). When compared with the solos group, the differences are found exclusively in pathologies such as angina pectoris or embolism and the percentage of monocytes. In the case of women (Table S3 available in Supplementary Materials), the differences found with respect to the total of the individuals are confirmed, and even new variables appear with differences between the depressed individuals group and the solos group with respect to the group of “solos and depressed individuals”, such as sleep or physical activity (Table S3a–c available in Supplementary Materials).

### 3.5. Genetic Association

With respect to the SNPs, we observed how, except rs2487999, the SNPs that had an effect on total loneliness were confirmed by dividing the sample into four groups (Table S8 is available in Supplementary Materials S4). Mutations in rs2154110 are the only ones in which a significant OR value is observed when considering the control group with respect to the other three groups. In the case of the “solos and depressed individuals” group, both the two SNPs (rs2154110 and rs4902100) encoding SYT16 as rs6265 (BDNF) appear with a significant OR.

### 3.6. Multivariable Analysis

#### Decision Trees

Decision trees were built considering the four groups in the reduced database. Although a tree was obtained with acceptable accuracy (52.1%), it failed in the classification of the group of “solos and depressed individuals” and was therefore discarded. When using the total database, the obtained tree has a precision value (52.5%), similar to that of the reduced database, and the correct classification percentage of the group “solos and depressed individuals” amounts to 41.3% but is not considered acceptable (data not shown).

## 4. Discussion

The aim of this paper is to analyze the role that loneliness plays in the state of health of lonely people in terms of different genetic, cognitive, biochemical, and lifestyle factors.

### 4.1. Characteristics of Lonely People

In the sample selected, it was observed that the demographic profile obtained in people with loneliness refers to men, with an average age of 50–60 years, who live in localities with a medium number of inhabitants (10–500), and who are overweight and short. Regarding sex, research examining the prevalence of loneliness among men and women reports conflicting results across all age groups [48]. The meta-analysis carried out by Mahon et al. (2006) [84] found that 19 studies did not report sex differences, 9 found that men were more lonely, and 2 found that women were more lonely. Antonucci’s research (2001) [85] suggests that men might feel greater loneliness by the mode of social relationship. Women would have more diverse social networks while men tend to focus more on intimate relationships with their partner. According to our regression model, it is men who show the greatest loneliness, and only 17.78% were widowed, single, separated, or divorced. 

Therefore, the association of living together with loneliness seems to be too simplistic. There are more parameters to consider, especially in elderly people among whom there can be cohabitations of several decades.

Regarding the main models on age and loneliness, our population conforms more to the model of Luhmann and Hawkley, finding the highest levels of loneliness among the 50–60 age group and no significant differences in the rest of the age ranges. These results break the widespread idea that loneliness is linked to being old [86,87,88,89].

It is not surprising that loneliness is associated with a greater body weight, although the strong impact it has on height is striking. No association with BMI was obtained. It is clear that the older we are, the more height we lose. This decrease in height is a potential marker of health in the last stage of life [90]. Decreased height is associated with loss of health mainly by compression and/or curvature of the backbone [91], sarcopenia, and reduced bone density [92]. Thus, Jain et al. (2020) [93], in a study on a sample in Indonesia—Indonesians are some of the shortest individuals in the world—conclude that this decrease in height in old age is a biomarker of worsening health. 

In the regression analysis, the biological variables that appear with predictive power are hemoglobin (b = −0.233, t = −2.5, *p* = 0.012), RDW (b = 0.299, t = 2.708, *p* = 0.007), and neutrophils (b = −0.032, t = −2.445, *p* = 0.014).

In the present study, high levels of RDW are found along with low hemoglobin levels, which are associated with total loneliness. A strong association has been demonstrated between anemia and the phenotypic characteristics of fragility syndrome, such as sarcopenia, reduction of muscle strength, and mobility problems [94]. In the study by Chaves et al. (2002) [95], which was carried out in women over 70 years of age, it was observed that in the objective assessment of mobility using the SPS scale (summary performance score: walking on short routes, moving from sitting to standing, and standing balance), worse scores were obtained in patients with hemoglobin above or below 14 gr/dL. Some studies have analyzed the relationship between anemia and subclinical cognitive alterations such as executive function (planning, monitoring, and problem-solving), which is an early marker of disability in instrumental activities [96,97,98]. In that way, a cross-sectional study [99] evaluated the relationship between mild anemia (10–12 g/dL) in a cohort of 364 women (70–80 years) with MMSE > 24 [71], and anemic patients were found to have a four-times-higher risk of obtaining worse scores when performing specific cognitive tests. Meanwhile, another study [97], which included 1744 individuals older than 71 years of age, revealed that those who were anemic at the beginning had a cognitive impairment greater than 4 years of follow-up evaluated with the Pfeiffer’s Short Portable Mental Status Questionnaire [100].

High levels of RDW reflect increased anisocytosis, which is associated with chronic diseases [101] and chronic inflammation [102]. Although the underlying mechanisms are still unknown, there is growing evidence of a pathogenic association between fragility, age anemia, and inflammation [103]. Aging is accompanied by persistent low-grade inflammation, known as “inflammaging”, which leads to an immunosenescence [104]. For phagocytes, monocytes, neutrophils, and macrophages, in general, no changes in total number are observed when getting older [105], but alterations in its general functionality are. Specifically, in neutrophils, a lower phagocytic and chemotaxis capacity is observed, even increasing its number [106]. In the present study, a lower percentage of neutrophils associated with loneliness is observed, which could be related to a possible worse response to infections with which the physiological vulnerability of the individual would increase.

### 4.2. Genetic Association with Loneliness

Regarding total loneliness, a significant association has been observed for six SNPs—rs2487999, rs391300, rs6265, rs12335203, rs2154110, and rs4902100—with respect to the sample of 414 individuals analyzed (Table 3). From these SNPs, and considering their inheritance models, the RGS has been calculated, which is the first classification variable in the tree with respect to loneliness (Figure 1). 

rs2487999 corresponds to an SNP present in the OBFC1 gene (*Oligonucleotide/oligosaccharide binding fold containing 1*), also called SNT1, located in 10q24.3, which codes for the STN1 protein (*Suppressor of cdc ThirteeN 1*). This protein is part of the CST complex. The human CTC1-STN1-TEN1 (CST) complex binds to ssDNA and has been implicated in protecting genomic stability under replication stress. Liu et al. (2021) [107] show that the CST complex localizes to replication forks in response to their stalling and protects them from degradation by MRE11 nuclease.

Although in rs2487999 the presence of the alternative allele is missense, Scheller et al. (2020) [108], in a study on the association of telomere shortening with Alzheimer’s disease, observed again that AA/TT individuals present telomeres longer than GA/CT and GG/CC, with significant differences (*p* value: 2 × 10–25). 

In the present study, it has been found that those individuals with higher values in loneliness have a higher frequency of the TT genotype, which, according to the studies mentioned above, correspond to the longest telomeres. However, it should be noted that the length of telomeres has not been measured in the present study, and that, given the sample size of the study, the individuals with higher levels of loneliness and with the TT genotype correspond to approximately 1.3% of all the individuals analyzed.

rs6265 is an SNP of the BDNF gene, which involves the substitution of the amino acid valine (Val) for methionine (Met) at codon 66 (G196A). This mutation may reduce activity-dependent BDNF secretion and trafficking in cortical neurons.

Bedard et al. (2017) [109], in a study on 252 students, found no association between loneliness, measured with the UCLA Loneliness Scale, and rs6265 genotypes, although they did observe some moderation of rs6265, relating it to the self-perception of an effective person (self-efficacy). Individuals with low self-efficacy with a Met allele had more pronounced depression symptoms and experienced greater loneliness, while in individuals with elevated self-efficacy, the homozygotes for Val showed the highest scores of depression and loneliness. Thus, Val/Val individuals may be more sensitive to adverse life experiences such as loneliness and more willing to present depressive symptoms. These results were surprising as, traditionally, the presence of the mutation, Met allele, had been associated with lower neuroplasticity and higher levels of depression. Gao et al. (2017) [53], in a large study of GWAs with 10,760 Americans, failed to replicate any of the SNPs that had previously been selected with loneliness, including rs6265. In the present study, loneliness is associated with the presence of Met alleles, which could imply a reduction in the structural and functional connectivity of the hippocampus and prefrontal cortex, e.g., [110].

rs391300 is an SNP of the SRR gene (serine racemase). Its mutation, T > C, is responsible for a change in an intron. SRR generates D-serine forms from L-serine, being a coagonist of the NMDA receptor (*N*-methyl-D-aspartate). In the present study, significant differences were observed with respect to loneliness levels in carriers of the T(A) allele. These results extend to those of Girard et al. (2017) [111], who analyzed the association of SNPs with the progression of mild cognitive impairment (MCI) to Alzheimer’s. In their final model, they only identified rs391300 as a risk factor. Analysis of sex shows that this association is observed only in men.

rs12335203 is an SNP, whose alleles are C and T, present in an intron of the TERF1 gene. TERF1 is one of the proteins that is part of the sheltering complex that protects telomeres from degradation. In the work of Crocco et al. (2015) [112], the presence of the C allele was associated with longevity in the population of Southern Italy, although it was not confirmed in the population of Northern Italy. Crocco et al. argued that their association could be specific to the population. In the present research, the C allele has been associated with the highest levels of loneliness (0.018). These differences are found to be concentrated among women. 

rs2154110 and rs492100 are located in the 14q23.2 region, in an intron of the SYT16 gene. Both variants were identified in a study of association with telomere length in a healthy, long-lived population [113]. SYT16 is involved in vesicle transfer and exocytosis in nonneural tissues. In the present research, both polymorphisms appear in linkage imbalance (D 0.1888; R 0.978). In both cases, homozygotes have the highest levels of loneliness (SOLTOT). Moreover, in both cases, it is in men that significant differences are observed between homozygotes and heterozygotes in SOLTOT.

In general, six genetic markers have been identified that are associated with loneliness. All markers that have a statistically significant association are located in genes responsible for maintaining telomere length and BDNF. The results presented do not allow to establish whether there is a shortening or an elongation of the telomeres. Wilson et al. (2019) [114] conducted a study of the relationship between telomere shortening, loneliness, the parasympathetic system, and herpesvirus reactivation. They hypothesized that people alone will feel more stressed and will have a greater number of adverse inflammatory responses and greater sympathetic cardiac activation. They obtained as main results that those individuals with higher values of heart rate variability (HRV) and greater loneliness had shorter telomeres. The difference with the non-solo was 482.6 base pairs [114]. The identified SNPs could be part of the set of markers responsible for telomere shortening with stressors such as loneliness.

### 4.3. Is Loneliness a Predictor of Worse Health?

In order to achieve the third objective of this work, four groups were established: control group, solos group, depressed individuals group, and “solos and depressed individuals group”. The data for the samples in which the genetic analysis was performed were analyzed with the data from the full database, including those in which the genetic analysis was not performed, so that two databases were available: the database with genetic analysis (*n* = 414) and total database (999 individuals). Regarding the first database, the statistical analyses did not consider the group of depressed individuals due to its small sample size.

There are numerous publications on the negative effect of loneliness on health status. However, the present work points out that the combined effect of loneliness on depression is what predicts a worse state of health. Regarding all the pathologies consulted to the participants, in all of them, on average, the “solos and depressed individuals group” presents more pathological values. 

The presence of recurrent feelings of loneliness is associated with low levels of satisfaction with life [33]. Satisfaction with life is the result of an individual’s cognitive assessment of their living conditions and achievements, comparing it to their needs and expectations and taking into account their personal and sociocultural values [115]. In our study, when considering their levels of satisfaction with life and health, etc., it is the solos and the solos–depressed people who present lower values, with significant differences with respect to the control groups, in all aspects that have been studied except in spiritual life, where there are no significant differences between the four groups. These results suggest that these are different constructs. The same happens in the study by Pinto are Neri (2013) [116], who analyzed the factors associated with life satisfaction based on data from a sample of 2472 people over 65 years of age (mean age 72.2 years) without dementia in the study “Frailty in Elderly Brazilians—FIBRA”. They analyzed, sex, age, chronic diseases, self-reported memory problems, urinary incontinence, falls, grip strength, walking speed, and social involvement. The analyses showed independent associations between variables such as having three or more chronic diseases, self-reported memory problems, feelings of loneliness, low social involvement, urinary incontinence, low grip strength, falls in the previous year, low walking speed, and low satisfaction with life. It means that life satisfaction levels tend to decrease with advanced age, although age itself cannot be interpreted as a direct cause of increased or decreased satisfaction with life. This type of relationship is best explained by the physical and social conditions in which a person lives and that, with age, unfavorable living conditions often emerge or worsen (for example, chronic diseases, functional difficulties, etc.) [117].

Regarding cognitive function, a greater heterogeneity of results is observed. For example, in semantic and phonological verbal fluency or orientation, it was the group of the “solos and depressed individuals” who presented lower values and significant differences with respect to the rest of the groups. To the extent known to it, there are no similar works that have included this group, although other authors who analyzed the solos group [118] found that the decrease in verbal fluency was a significant predictor of loneliness. Though O’Luanaigh et al. (2012) [41] also reported a relationship between loneliness and verbal fluency, this result did not reach significance (*p* = 0.079) after controlling a series of demographic factors along with depression and social relations. Finally, Shankar et al. (2013) [45] found that increased loneliness was significantly associated with low baseline verbal fluency, but not in a 4-year follow-up. In addition, poor communication skills, poorer verbal fluency, and low interest in social contacts in people with depression can hinder conversations and meaningful relationships and, consequently, increase feelings of loneliness. Several studies report a relationship between deficits in both measures of verbal fluency (for example, [119]) in the presence of depression. In particular, semantic fluidity has been found to be associated with a greater deficit than phonological fluidity in depression [120], suggesting that depression may be especially associated with a semantic memory deficit. The paucity of studies and mixed results to date suggest more research needs to be conducted on the relationship between verbal fluency and loneliness, including the group of solos and depressed individuals.

In terms of memory, in the three-word delay recall task of the MMSE, individuals present a lower mean value compared to the depressed and do not present significant differences compared to the group of “solos and depressed individuals”, but when we compare the group of depressed to that of “solos and depressed individuals”, again it is these who have a worse performance of delay recall compared to the depressed individuals. Moreover, the same happens in the semantic memory task, evaluated with the Boston Naming Test, the results of which show that the “solos and depressed individuals” obtain a lower performance compared to the depressed ones. Our results support the findings of other authors who indicate that depression and loneliness negatively affect memory, among other capabilities [121], and, as stated above, Christensen et al. (1997) [119] suggest that depression may be associated with a deterioration of semantic memory.

In relation to attention, it was evaluated with the attention task in the MMSE. The results indicate that the “solos and depressed individuals” obtain worse results both when compared with the solos group and when compared with the depressed individuals group. Authors such as Schnittger et al. (2012) [115] and Tzang et al. (2015) [120] have found negative correlations between loneliness and performance in attentional tests.

Regarding the global cognitive function, there are only statistically significant differences in the total MMSE score in the solos group versus depressed individuals group, the solos group being the one with the worst overall performance. Loneliness can decrease the neural reserve through decreased dendritic arborization in the hippocampus and prefrontal cortex, resulting in the decrease in cognitive abilities such as orientation, attention, and memory [40]. Shankar et al. (2013) [45] found that greater loneliness predicted worse cognitive function. However, Zhong et al. (2017) [121] indicate that there may be a two-way relationship between loneliness and cognition. On the one hand, loneliness predicts the deficit of different cognitive abilities such as processing speed, memory, or verbal fluency and general cognitive functioning [41,46]. On the other hand, a low cognitive status can predict a reduction in social contact and in the size of the social network, as well as difficulties in maintaining friendships and communicating with others, which in turn could lead to higher levels of loneliness [43,95]. Thus, loneliness could also be a consequence of impaired cognitive functioning [46,122]. Furthermore, interventions targeting loneliness also show a beneficial effect on cognitive functioning [123]. The causal relationship between loneliness, depression, and cognitive function needs to be further investigated.

The results in relation to sleep patterns are very significant. The “solos and depressed individuals” sleep fewer hours at night, wake up feeling rested on less days, and have more days with difficulty falling asleep or when they wake up too early. Moreover, in the same way, it is the individuals of this group who perform less physical activity. However, they only present significant differences when compared to the control and solos groups. The same results are observed in the group of women. Goossens et al. (2015) [55] argue, within the evolutionary theory of loneliness, that loneliness can lead to sleep problems, which can increase blood pressure. Although the present study confirms the difficulty of sleeping among those people who have loneliness and depression, they do not have problems of tension but blood circulation. It has been proposed that hypervigilance produces high stress and reduces self-regulation capacities, making it difficult to promote health (for example, physical activity) in solitary people [9,82]. Recently, Jin et al. (2022) [124] have established a link between sleep patterns and telomeres. In a study of 238 participants, aged between 55 and 88 years, they observed a consistent association between short sleep duration, higher latency, and a faster telomere-shortening rate. Regarding the possible biochemical mechanisms that link sleep quality and telomeres, they propose inflammation, oxidative stress, cortisol secretion, and/or increased sympathetic activity [125].

All the data collected in the present work point to a combination of depression and loneliness for the prognosis of a worse state of health, worse cognitive status, a perception of their worse quality of life, worse quantity and quality of sleep, and with less physical activity.

With regard to these results, we must first emphasize the importance of eliminating loneliness as a prognostic factor for a worse state of health in the elderly population, as has been pointed out in numerous studies [81,126]. Loneliness is even associated with increased fragility, understood as the deterioration of multiple related physiological systems and a greater vulnerability to different stressors [127]. In the present work, the results do not support this belief, since the “solos” have a state of health similar to the control group individuals.

Loneliness has been considered as a marker of a worse state of health, as stated, for example, in the work of De la Torre-Luque et al. (2021) [128]. This paper sought to establish the role of loneliness in metabolic deregulation in elderly people with depression. Three groups were established: control group (without depression), depressed individuals group, and depressed–solos individuals group. They conclude that due to the role of loneliness in depression accompanied by metabolic alterations, it is necessary to monitor loneliness in older people due to its impact on mental and physical health. These recommendations are due, among others, to the observation of an increased metabolic risk in the group of “solos and depressed individuals”, especially in the case of women. In this work, the solos group has not been valued. In the present research, it is also the group of “solos and depressed individuals” who have a worse state of health; yet the group of the solos also present a state of health very similar to the control group, and therefore, it is ruled out that loneliness in itself implies a worse state of health. That is, loneliness is a prognostic factor for poorer health in people who have depression.

This discrepancy with other studies may be due to the difference between loneliness and social isolation. Social isolation reflects the objective characteristics of an individual’s social situation and refers to the absence of social contacts and social relations [6]. In those studies in which both parameters have been used, such as their association with mortality, only social isolation has been found to have an effect [129,130,131]. Thus, loneliness could be associated with a worse state of health when it could be social isolation. On the other hand, it is essential to clearly assess loneliness. It should not be valid to ask a single question when the volunteer is asked if they feel alone or how often they feel alone. It is necessary to use valid and reliable evaluation tools for the group of people being evaluated, tools that clearly identify individuals alone, and to distinguish them from those who are isolated or depressed and lonely.

Secondly, the combination of loneliness and depression leads to a worse state of health. The strong relationship between loneliness and depression is well-documented, and it is accepted that, over time, high levels of loneliness predict depression [132,133,134,135] due to its association with numerous sociodemographic and psychosocial predictors. However, there are already authors such as Abdellaoui et al. (2018) [136] who insist that they are two different “constructs”. Loneliness is usually defined in relation to discrepancies between the actual and desired social contact [5], and depression refers to a mood disorder [137]. The results presented in the present work allow to define a third construct that would be constituted by the group that present both loneliness and depression together. It is difficult to determine whether loneliness is before, contemporary to, or after depression. It is even difficult to indicate whether one is a consequence of the other, but they do constitute a distinct group of the lonely and the depressed. Arguments in favor of defining this group as a group with its own entity are its obviously worst generalized health status. For example, it is the depressed individuals group and the “solos and depressed individuals group” who present a greater number of diseases.

**Figure 3:**
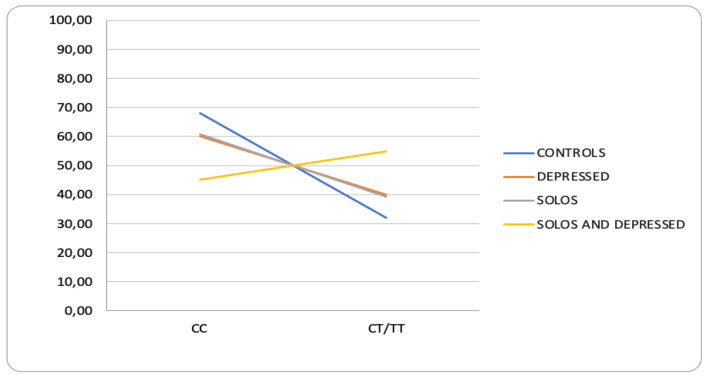
Representation of the frequencies of the genotypes of rs6265 (BDNF) with respect to the groups: controls, depressed, solo, and solo and depressed.

Regarding the SNPs analyzed, although a common SNP appears, rs2154110, the truth is that they present a set of specific SNPs in each group. Thus, for instance, rs6265 (BDNF) only has an association in the “solos and depressed individuals” group and not in the solos group or in the depressed individuals group (Table S4 available in Supplementary Materials). Against the evolutionary models of loneliness, in which it is proposed that the presence of certain genetic variants would be responsible for a greater sensitivity to socioenvironmental factors and would lead to higher levels of loneliness [60], it seems that alleles of the SNPs related to changes in telomere length, such as those of SYT16 and BDNF, would play a more important role in the group of “solos and depressed individuals”. Several studies have demonstrated the association between depression and telomere shortening [138,139], although this association with the alleles of the SNP rs2487999 or rs12335203, identified among the solos group, is not confirmed in the group of “solos and depressed individuals”. The results presented by Bedard et al. (2017) [109], in which they propose that the presence of Val/Val genotypes makes people more vulnerable to experiences of loneliness, have already been discussed. In the present work, the group of “solos and depressed individuals” is where the highest frequencies of genotypes C/T and T/T appear (Figure 3). These results, unlike Bedard et al. (2017) [109], would be compatible with the neurotrophic hypothesis of depression [140,141,142], according to which a reduced secretion of BDNF could lead to increased vulnerability to depression [143]. Losenkov et al. (2020) [144] found an association between rs6265 genotypes and the severity of depression in depressed patients.

Regarding SYT16, and the role of telomeres in depression, there is the hypothesis that individuals with depression present a faster rate of shortening [145]. In a meta-analysis of 56 studies, depression, along with other psychiatric diseases, was associated with shorter telomeres [146]. Some studies have not obtained significant differences; however, this seems to be due to the origin of the analyzed sample. However, for example, in some studies no differences have been found in cells of the hippocampus or the nucleus accumbens [111].

It is possible that this telomere shortening in the depressed ones acts through BDNF. Nunes et al. (2018) [147] consider depression as a premature aging syndrome characterized by low levels of BDNF. It is in the depressed and solos group where a higher percentage of BDNF genotypes with a Met allele have been found, as well as the association with genes related to telomere shortening. The presented results point to a mediating role of loneliness through telomeres and BDNF, having as an effect a worse generalized state of health.

On the other hand, the existence of the “solos and depressed individuals” group as a distinct entity is not incompatible with the established idea that loneliness increases depressive symptoms [148,149]. In this sense, the work of Maarsingh et al. (2018) [150] has shed interesting light on it. They have developed a model to evaluate depression in the elderly, in which their main predictors are: loneliness, anxiety symptoms, and the severity of depression. The results obtained in the present work would be in line with those of Maarsingh, although the severity of depression has not been assessed, and anxiety presents similar levels between the depressed and the group of “solos and depressed individuals”.

Regarding the physiological pathways that connect depression with loneliness, there are different hypotheses with contradictory results. Some point to the role of the HPA axis (hypothalamic–pituitary–adrenal) through the action of cortisol, although recent research has not allowed to confirm it [151]. For metabolic diseases, the possibility of inflammatory deregulation is raised [128]. The role of telomeres in relation to the HPA axis [152,153] or inflammation [125] has also been studied. In the present work, the role of telomere length along with BDNF, as an intermediary between loneliness and depression, and its relationship with a worse state of health are discussed. Given the worst general health condition, involving very different pathologies, new research is needed to assess more parameters related to biochemical stressors to establish the pathways of the interaction of loneliness with health status.

The relationship between depression and loneliness is complex. Most importantly, loneliness and depression are common in this group of people [86,154], suggesting that loneliness and depression problems are important among adults and seniors. Depression in the elderly may be underestimated, with loneliness being a key risk factor in relation to health status from a global perspective. Effective holistic prevention and intervention strategies aimed at reducing loneliness, as proposed by Rødevand et al. (2021) [155], could have beneficial effects on quality of life and psychosocial functionality, as well as protective effects against the development of other diseases among people suffering from depression.

Our work has a number of strengths. One of them is that it is based on a representative sample of the Spanish population (See Study Design, Section 2.1) In addition, it has a novel approach to highlight what it involves considering loneliness as a feeling associated with people’s lives and not necessarily as something pathological, given that loneliness as a source of pathology is strongly rooted in numerous works. Another strong point is the number of variables considered (See Measurements, Section 2.3) and the range of possible factors to consider (psychological, lifestyle, biochemical, genetic, etc.). A relevant and novel role is the genetic factor, which has revealed the activity of telomerase as an element to consider in future studies.

The present study also has a number of limitations. The first one is the transversal nature of the data, which limits the ability to establish causality in the relationships between the variables of interest. In addition, it would have been desirable to have a greater sample of individuals with depression without loneliness. The results of the study have not allowed us to draw conclusions about the direction of the relationship between loneliness and depression or to establish what the biochemical and physiological mechanisms that support this association are. Nonetheless, longitudinal studies could help to elucidate the direction of this relationship.

In addition, measurement bias cannot be ruled out in the evaluation of depressive symptoms, satisfaction with life, and other parameters analyzed through a self-report questionnaire. Moreover, although perceived loneliness is associated with personality characteristics, especially neuroticism [135], and some researchers have suggested that loneliness is a trait, there may be a solitary personality characterized by high neuroticism, low extraversion, deficit in the processing of social information, and poor social skills [156]; we could not study this variable since this information was not collected in the original study. Thus, this it is worth investigating in the future.

## 5. Conclusions

In conclusion, it was observed that the demographic profile obtained in people with loneliness refers to men, with an average age of 50–60 years, who live in localities with a medium number of inhabitants (10–500), and who are overweight and short. Six genetic markers have been identified that are associated with loneliness. All markers are in genes responsible for maintaining telomere length and BDNF.

When we considered depressed state and loneliness, we obtained a worse state of health, a worse cognitive status, a perception of their worse quality of life, worse quantity and quality of sleep, and less physical activity. We concluded that the “solos” have a state of health like that of the control group individuals. This breaks with the stereotypes that currently exist about loneliness. In fact, loneliness is a prognostic factor for poorer health in people who have depression. It proposes the role of telomere length together with BDNF as an intermediary between loneliness and depression. 

More longitudinal research is needed if we are to understand the independent relationships between loneliness and depression, as well as more research into the associations between depression and the types of loneliness (emotional and social) over time.

## Figures and Tables

**Table 1:** Comparisons of means or counts and standard deviation, and *p*-value. Only those comparisons that are statistically significant (*p* ≤ 0.05) are collected.

Variable	Levels	No Loneliness	With Loneliness	*p* Value
Marital Status	Single Married Living with partner Widower Separate Divorced	1:6 2:96 3:1 4:16 5:1 6:4	1:6 2:138 3:1 4:33 5:4 6:9	0.042
Level of satisfaction with your life in general	Scale from 0 to 10 (0 means the lowest level of satisfaction and 10 the highest level of satisfaction)	8.19 (1.25)	7.46 (1.46)	<0.001
Level of satisfaction with your state of health	Scale from 0 to 10 (0 means the lowest level of satisfaction and 10 the highest level of satisfaction)	7.40 (1.56)	6.98 (1.74)	0.026
Level of satisfaction with personal relationships	Scale from 0 to 10 (0 means the lowest level of satisfaction and 10 the highest level of satisfaction)	8.43 (1.16)	8 (1.28)	0.03
Nº of times per month that you participate in cultural activities (eg., going to the cinema, theater, exhibitions)	0 Never 1. 1–3 times per month 2. 1–2 times a week 3. 3–4 times a week 4. 5 or more times per week	0:72 1:8 2:18 3:12 4:19	0:127 1:5 2:19 3:19 4:20	0.009
Level of satisfaction with the way you spend your free time	Scale from 0 to 10 (0 means the lowest level of satisfaction and 10 the highest level of satisfaction)	8.96 (11.44)	6.73 (1.75)	0.008
Level of satisfaction with friendship relationship	Scale from 0 to 10 (0 means the lowest level of satisfaction and 10 the highest level of satisfaction)	7.83 (1.50)	6.79 (1.85)	<0.001
Sleeping difficulties	Never Some days Several days Most days Every day	1:90 2:20 3:6 4:9 5:4	1:94 2:52 3:17 4:17 5:8	0.014
Sleep quality	Sum of the items: difficulty falling asleep, waking up several times, and waking up too early	5.58 (2.74)	6.44 (3.52)	0.015
Circulation problems (excluding varicose veins), intermittent claudication	1 Yes 0 No	0:115 1:14	0:152 1:38	0.024
Back pain	Scale from 0 to 10 (0 means the lowest level of satisfaction and 10 the highest level of satisfaction)	0.39 (1.43)	0.85 (2.32)	0.027
3 word Memory	0 words 1 words 2 words 3 words	0:2 1:11 2:34 3:82	0:8 1:21 2:64 3:96	0.027
Writing	0 1	0:0 1:129	0:9 1:182	0.002
Animal verbal fluency		19.59 (6.38)	18.15 (5.99)	0.043
MCHC (g/dL)		33.18 (0.98)	32.91 (1.23)	0.043
RDW (%)		13.57 (0.73)	13.85 (1.16)	0.007

MCHC (mean corpuscular hemoglobin concentration), RDW (Red Blood Cell Distribution Widht).

**Table 2:** Description of SNPs. Data from GWAS Catalog (https://www.ebi.ac.uk/gwas/home accessed on 1 September 2022). Calculation of the Hardy–Weinberg equilibrium.

SNPs	Gene or Closest	Position (GRCh38.p13)	Genotype	N	Frequency	Chi-Squared	*p* Value
rs10509637	TNKS2	10:91827975	GG	7	0.017	0.2109	0.900
			AG	102	0.246		
			AA	305	0.737		
rs10792832	RNU6-560P	11:86156833	GG	196	0.473	0.1801	0.914
			AG	175	0.423		
			AA	43	0.104		
rs10904887	TRDMT1	10:17146642	TT	110	0.266	0.8926	0.640
			CT	216	0.522		
			CC	88	0.213		
rs10904896	TRDMT1	10:17183827	GG	110	0.266	0.8926	0.640
			AG	216	0.522		
			AA	88	0.213		
rs10936599	MYNN	3:169774313	TT	20	0.048	0.0357	0.982
			CT	145	0.350		
			CC	249	0.601		
rs12335203	TERF1	8:73041384	TT	80	0.193	0.121	0.941
			CT	200	0.483		
			CC	134	0.324		
rs12638862	TERC	3:169759718	GG	21	0.051	0.7162	0.699
			AG	158	0.382		
			AA	235	0.568		
rs12696304	TERC	3:169763483	GG	23	0.056	1.4247	0.490
			CG	168	0.406		
			CC	223	0.539		
rs1317082	MYNN	3:169779797	GG	21	0.051	0.0009	1.000
			AG	144	0.348		
			AA	249	0.601		
rs16847897	LRRC31	3:169850328	GG	242	0.585	2.6376	0.267
			CG	141	0.341		
			CC	31	0.075		
rs16859140	TMPRSS7	3:112073747	TT	241	0.582	0.4699	0.791
			CT	153	0.370		
			CC	20	0.048		
rs182549	LPH	2:135859184	TT	79	0.191	0.4461	0.800
			CT	196	0.473		
			CC	139	0.336		
rs1990622	TMEM106B	7:12244161	GG	71	0.171	0.8252	0.662
			AG	190	0.459		
			AA	153	0.370		
rs2066276	TNKS2	10:91797862	TT	92	0.222	1.6653	0.725
			CT	220	0.531		
			CC	102	0.246		
rs2154110	SYT16	14:62086065	TT	225	0.543	0.1995	0.905
			GT	158	0.382		
			GG	31	0.075		
rs2293607	TERC	3:169764547	TT	249	0.601	0.1687	0.919
			CT	146	0.353		
			CC	19	0.046		
rs2487999	STN1	10:103900068	TT	4	0.001	0.0353	0.983
			CT	70	0.169		
			CC	340	0.821		
rs2735940	TERT	5:1296371	GG	90	0.217	0.1385	0.933
			AG	202	0.488		
			AA	122	0.295		
rs2736100	TERT	5:1286401	CC	105	0.254	1.4589	0.482
			AC	219	0.529		
			AA	90	0.217		
rs2967374	RN7SKP190	16:82176256	GG	247	0.597	0.9295	0.628
			AG	141	0.341		
			AA	26	0.063		
rs3764650	ABCA7	19:1046521	TT	314	0.758	2.3624	0.307
			GT	97	0.234		
			GG	3	0.007		
rs3772190	MYNN	3:169782699	GG	249	0.601	0.0357	0.982
			AG	145	0.350		
			AA	20	0.048		
rs3784929	KARS1	16:75643129	GG	6	0.014	3.434	0.180
			AG	59	0.143		
			AA	349	0.843		
rs3818361	CR1	1:207611623	GG	290	0.700	0.0885	0.957
			AG	112	0.271		
			AA	12	0.029		
rs391300	SSR	17:2312964	TT	72	0.174	0.1071	0.948
			CT	197	0.476		
			CC	144	0.348		
rs4633	COMT	22:19962712	TT	102	0.246	0.4578	0.795
			CT	200	0.483		
			CC	112	0.271		
rs4902100	SYT16	14:62083101	GG	30	0.072	0.4484	0.799
			AG	153	0.370		
			AA	231	0.558		
rs6265	BDNF	11:27658369	TT	17	0.041	0.1939	0.908
			CT	141	0.341		
			CC	256	0.618		
rs755017	ZBTB46	20:63790269	GG	7	0.017	1.2897	0.525
			AG	75	0.181		
			AA	332	0.802		
rs7675998	TOMM22P4	4:163086668	GG	234	0.565	0.0097	0.995
			AG	154	0.372		
			AA	26	0.063		
rs7726159	TERT	5:1282204	CC	155	0.374	1.4647	0.481
			AC	206	0.498		
			AA	53	0.128		
rs8105767	ZNF257	19:22032639	GG	23	0.056	1.4247	0.490
			AG	168	0.406		
			AA	223	0.539		
rs9420907	STN1	10:103916707	CC	6	0.014	1.0382	0.595
			AC	107	0.258		
			AA	301	0.727		
rs429358	APOE	19:44908684	TT	352	0.850	18.083	0.000
			CT	52	0.126		
			CC	10	0.024		
rs7412	APOE	19:44908822	TT	4	0.010	2.4417	0.295
			CT	49	0.118		
			CC	361	0.872		
APOE	E2E2	E2E2	E2E2	4	0.010		
	E2E3	E2E3	E2E3	41	0.099		
	E2E4	E2E4	E2E4	8	0.019		
	E3E3	E3E3	E3E3	306	0.741		
	E3E4	E3E4	E3E4	44	0.107		
	E4E4	E4E4	E4E4	10	0.024		

**Table 3:** Analysis of association of SNPs with levels of loneliness (SOLTOT) based of lineal regression. The models with significant p and the lowest Akaike’s Information Criterion (AIC) and Bayesian Information Criterion (BIC) are presented.

SNP	Model	Genotype	n	Response Mean (s.e.)	Difference (95% CI)	*p*-Value
rs12335203	Log-additive	CC	95	2.41 (0.18)	−0.31 (−0.57–−0.05)	0.018
		CT	161	1.99 (0.13)		
		TT	64	1.81 (0.2)		
rs391300	Dominant	CC	105	2.49 (0.17)	0	
		CT/TT	214	1.87 (0.11)	−0.61 (−0.99–−0.23)	0.0018
rs2487999	Log-additive	CC	259	1.98 (0.1)	0.47 (0.05–0.88)	0.029
		CT	57	2.44 (0.25)		
		TT	4	3 (0.41)		
rs2154110	Overdominant	TT/GG	199	2.29 (0.12)	0	
		GT	121	1.73 (0.14)	−0.56 (−0.93–−0.19)	0.0035
rs4902100	Codominant	AA	183	2.21 (0.13)	0	
		AG	117	1.74 (0.15)	−0.45 (−0.83–−0.07)	0.0061
		GG	20	2.85 (0.25)		
rs6265	Codominant	CC	197	1.88 (0.11)	0	
		CT	113	2.27 (0.17)	0.39 (0.01–0.76)	0.0002
		TT	10	3.9 (0.5)	2.02 (1.00–3.05)	

**Table 4:** Regression coefficients for the loneliness (SOLTOT) model.

		Total			
Variable	Levels	Estimate	SE	T	*p* Value
Sex	Men Women	−1.093	0.268	−4.071	<0.0001 **
Age	50–59 years 60–69 years 70–79 years 80–89 years	−0.819	0.318	−2.573	0.011 *
Number of inhabitants in participants’ usual municipality	Less than 10,000 pp. 10,000–100,000 pp. 100,000–500,000 pp. More than 500,000 pp.	0.694	0.248	2.801	0.005 **
Height	Between 1.31 m and 1.87 m	0.021	0.008	2.560	0.011 *
Weight	Between 42 kg and 127.80 kg	−6.181	1.238	−4.993	<0.0001 **
Life satisfaction	Scale from 0 to 10 (0 means the lowest level of satisfaction and 10 the highest level of satisfaction)	−0.330	0.076	−4.323	<0.0001 **
Sleep quality	Sum of the items: difficulty falling asleep, waking up several times, and waking up too early	0.064	0.033	1.950	**0.051**
Medicine consumption	Between 0 and 14 different medicines	0.032	0.015	2.094	0.036 *
Back pain	Scale from 0 to 10 (0 means the lowest level of satisfaction and 10 the highest level of satisfaction)	0.145	0.049	2.940	0.003 **
Cholesterol (mg/dL)	Between 103 mg/dL and 356 mg/dL	−0.006	0.003	−1.888	0.059
Iron (ug/dL)	Between 15 µg/dL and 188 µg/dL	0.006	0.004	1.560	0.119
Hemoglobin (g/dL)	Between 6.40 g/dL and 18.20 g/dL	−0.233	0.093	−2.501	0.012 *
RDW (%)	Between 11.80 and 21.00	0.299	0.111	2.708	0.007 **
Platelets (× 1000/mm^3^)	Between 74,000/mm^3^ and 520,000/mm^3^	−0.004	0.002	−1.905	0.057
Neutrophils	Between 1.1 and 11.29	−0.032	0.013	−2.445	0.014 *
Eosinophil ratio	Between 0 and 12.2	−0.105	0.063	−1.672	0.095
RGS	Between 0 and 10	0.195	0.043	4.544	<0.0001 **
Attentional level	Scale from 1 to 6 (0 means the lowest level of attention and 10 the highest level of attention)	−0.335	0.216	−1.553	0.120
MMSE	0–23 score (moderate-to-severe cognitive impairment) 24–26 (mild cognitive impairment) 27–30 (healthy cognitive performance)	2.433	1.086	2.240	0.025 *
Learning potential score (Auditory Verbal Learning Test)	Between 1 and 17	-0.071	0.043	−1.646	0.100
3 Word Memory	0 words 1 words 2 words 3 words	−0.430	0.169	−2.539	0.011 *
Last letter cancelled score (Cancelation Test)	Between 1 and 70	−0.026	0.009	−2.981	0.003 **

MMSE: Mini-Mental State Examination; RDW: red cell distribution width; GRS: genetic risk score. * *p* < 0.05, ** *p* < 0.01.

## Data Availability

Data al individual level is available upon request to first autor.

## Supplementary Materials

The following supporting information can be downloaded at: https://www.mdpi.com/article/10.3390/ijerph192315456/s1, Table S1. Comparison of means between groups. Table S2. Comparison of means between groups in men. Table S3. Comparison of means between groups in women. Table S4. Logistic regression analysis SNPs and groups.
