# Supplementary material for: Loneliness, Depression, and Genetics in the Elderly: Prognostic Factors of a Worse Health Condition?
Source: Int J Environ Res Public Health. 2022 Nov 22;19(23):15456. doi: 10.3390/ijerph192315456 (PMC9739711; doi:10.3390/ijerph192315456)
Supplement: Supplementary file 1 [file ijerph-19-15456-s001.zip › Supplementary Material S1. Tabla S1. Comparison of means between groups.pdf]

Tabla S1. Comparison of means between depressed individuals and solos groups.							
	Groups	Number	Median	standard deviation	t	Degrees of freedom	P-value
Health status	Depressed	64	3,64	0,72	-0,77	495	.441
	Solos	433	3,71	0,68	-0,74	80,38	
Circulation problems (excluding varicose veins). intermittent claudication	Depressed	66	0,18	0,39	0,35	507,00	0,365
	Solos	443	0,16	0,37	0,33	83,66	
Osteoarthritis	Depressed	66	0,39	0,49	0,97	508,00	0,167
	Solos	444	0,33	0,47	0,94	83,74	
Arthritis or rheumatism	Depressed	66	0,17	0,38	0,38	508,00	0,352
	Solos	444	0,15	0,36	0,37	83,32	
Asthma	Depressed	66	0,09	0,29	1,69	507,00	0,099
	Solos	443	0,04	0,20	1,30	74,78	
Anxiety	Depressed	66	0,33	0,48	5,92	508,00	<.001
	Solos	444	0,09	0,28	4,09	72,03	
Osteoporosis	Depressed	66	0,09	0,29	-0,58	508,00	0,283
	Solos	444	0,11	0,32	-0,62	90,15	
Chronic back pain	Depressed	66	0,24	0,43	0,97	508,00	0,167
	Solos	444	0,19	0,39	0,91	81,89	
Urinary tract problems (prostate. bladder)	Depressed	66	0,06	0,24	-1,10	508,00	0,097
	Solos	444	0,10	0,31	-1,31	99,01	
During the last 4 weeks. to what extent has any type of pain made it difficult for you to do your usual work (paid work and domestic or care work) or your leisure activities?	Depressed	66	1,70	1,14	0,02	508	.982
	Solos	444	1,69	1,10	0,02	83,99	
Headache	Depressed	11	1,27	2,53	0,72	94	.475
	Solos	85	0,82	1,87	0,57	11,46	
**Feet pain**	Depressed	10	0,30	0,95	-1,43	95	**.007**
	Solos	87	1,49	2,61	-2,91	29,08	
**Creatinine (mg/dL)**	Depressed	62	0,78	0,14	-2,66	485	**.000**
	Solos	425	0,86	0,23	-3,81	116,75	
Monocyte percentage	Depressed	45	6,99	1,22	-0,87	354	.387

	Solos	311	7,14	1,12	-0,81	55,27	
**Age**	Depressed	66	61,73	8,79	-2,22	508	**.016**
	Solos	444	64,64	10,09	-2,46	92,43	
Level of satisfaction with your life in general	Depressed	48	6,88	1,45	-0,87	390	.383
	Solos	344	7,04	1,17	-0,74	55,84	
Level of satisfaction with your state of health	Depressed	55	6,27	1,42	-1,86	417	.064
	Solos	364	6,66	1,43	-1,87	71,56	
Level of satisfaction with the achievements you are currently achieving in life	Depressed	46	6,96	1,32	0,56	402	.573
	Solos	358	6,84	1,38	0,59	58,50	
Level of satisfaction with personal relationships	Depressed	32	7,31	1,36	-0,05	335	.963
	Solos	305	7,32	0,98	-0,04	34,50	
Level of satisfaction with how safe and secure you feel	Depressed	34	7,29	0,80	0,96	357	.336
	Solos	325	7,09	1,19	1,33	49,92	
Level of satisfaction with their feeling of belonging to a community or group of people	Depressed	42	6,98	1,47	0,00	387	.997
	Solos	347	6,98	1,34	0,00	49,60	
**Number of diseases**	Depressed	65	4,34	2,26	3,26	501,00	**<.001**
	Solos	438	3,47	1,95	2,93	78,78	
CO_task cancellation hits made	Depressed	65	18,51	5,22	1,54	502	.125
	Solos	439	17,31	5,93	1,69	90,32	
Semantic fluency	Depressed	66	19,83	5,40	1,78	507	.076
	Solos	443	18,55	5,49	1,80	86,29	
Phonological fluency	Depressed	66	12,06	4,36	1,48	507	.141
	Solos	443	11,05	5,29	1,70	95,96	
Number of objects identified	Depressed	66	15,70	4,33	1,27	508	.205
	Solos	444	14,89	4,90	1,39	91,60	
**Orientation**	Depressed	66	9,91	0,29	1,40	508	**.047**
	Solos	444	9,82	0,48	2,00	125,96	

Attention and calculation	Depressed	66	4,82	0,46	0,58	508	.565
	Solos	444	4,78	0,55	0,66	95,00	
**3 Words Memory**	Depressed	66	2,70	0,55	3,30	508	**.000**
	Solos	444	2,37	0,77	4,18	105,96	
**Global cognitive function**	Depressed	66	29,14	0,99	2,75	508	**.000**
	Solos	444	28,62	1,47	3,67	112,71	

Tabla 5b. Comparison of means between depressed individuals and "solos and depressed" groups.

	Groups	Number	Median	standard deviation	t	Degrees of freedom	P-value
**Health status**	Depressed	64	3,64	0,72	2,341	207	**0,02**
	Solos and depressed	145	3,37	0,78	2,416	129,976	
Circulation problems (excluding varicose veins). intermittent claudication	Depressed	66	0,18	0,39	-1,245	214	0,097
	Solos and depressed	150	0,26	0,44	-1,307	139,647	
Osteoarthritis	Depressed	66	0,39	0,49	-1,169	214	0,121
	Solos and depressed	150	0,48	0,50	-1,177	126,344	
Arthritis or rheumatism	Depressed	66	0,17	0,38	-1,248	212	0,095
	Solos and depressed	148	0,24	0,43	-1,315	141,952	
Asthma	Depressed	0,09	0,29	0,29	-0,505	213	0,307
	Solos and depressed	0,11	0,32	0,32	-0,524	136,31	
Anxiety	Depressed	66	0,33	0,48	-1,109	214	0,131
	Solos and depressed	150	0,41	0,49	-1,126	128,873	
**Osteoporosis**	Depressed	66	0,09	0,29	-2,191	214	**0,007**
	Solos and depressed	150	0,21	0,41	-2,5	172,214	
**Chronic back pain**	Depressed	66	0,24	0,43	-1,613	214	**0,048**
	Solos and depressed	150	0,35	0,48	-1,68	137,087	
**Urinary tract problems (prostate. bladder)**	Depressed	66	0,06	0,24	-2,135	213	**0,006**
	Solos and depressed	149	0,17	0,38	-2,513	185,779	

**During the last 4 weeks. to what extent has any type of pain made it difficult for you to do your usual work (paid work and domestic or care work) or your leisure activities?**	Depressed	66	1,70	1,14	-1,877	214	**0,047**
	Solos and depressed	150	2,05	1,35	-2,004	145,686	
Headache	Depressed	11	1,27	2,53	-0,617	56	0,54
	Solos and depressed	47	1,83	2,73	-0,647	15,915	
Feet pain	Depressed	10	0,30	0,95	-1,396	55	0,168
	Solos and depressed	47	1,55	2,79	-2,48	43,641	
Creatinine (mg/dL)	Depressed	62	0,78	0,14	-0,986	197	0,325
	Solos and depressed	137	0,81	0,18	-1,089	151,255	
Monocyte percentage	Depressed	45	6,99	1,22	1,481	136	0,141
	Solos and depressed	93	6,67	1,17	1,462	84,274	
**Age**	Depressed	66	61,73	8,79	-1,843	214	**0,046**
	Solos and depressed	150	64,56	11,04	-2,012	154,106	
Level of satisfaction with your life in general	Depressed	48	6,88	1,45	0,895	176	0,372
	Solos and depressed	130	6,65	1,47	0,899	84,632	
Level of satisfaction with your state of health	Depressed	55	6,27	1,42	1,766	184	0,079
	Solos and depressed	131	5,81	1,72	1,907	121,436	
**Level of satisfaction with the achievements you are currently achieving in life**	Depressed	46	6,96	1,32	2,643	170	**0,005**
	Solos and depressed	126	6,27	1,57	2,87	94,832	
Level of satisfaction with personal relationships	Depressed	32	7,31	1,36	1,115	134	0,267
	Solos and depressed	104	7,02	1,29	1,084	49,393	

**Level of satisfaction with how safe and secure you feel**	Depressed	34	7,29	0,80	2,087	146	**0,005**
	Solos and depressed	114	6,72	1,54	2,887	108,178	
Level of satisfaction with their feeling of belonging to a community or group of people	Depressed	42	6,98	1,47	1,149	166	0,252
	Solos and depressed	126	6,65	1,63	1,207	76,918	
**Number of diseases**	Depressed	65	4,34	2,26	-2,267	208	**0,012**
	Solos and depressed	145	5,17	2,52	-2,364	136,568	
**CO_task cancellation hits made**	Depressed	65	18,51	5,22	2,487	211	**0,007**
	Solos and depressed	148	16,17	6,74	2,744	155,732	
**Semantic fluency**	Depressed	66	19,83	5,40	2,45	214	**0,015**
	Solos and depressed	150	17,63	6,35	2,61	144,938	
**Phonological fluency**	Depressed	66	12,06	4,36	1,774	214	**0,047**
	Solos and depressed	150	10,61	5,96	1,998	166,649	
**Number of objects identified**	Depressed	66	15,70	4,33	2,047	214	**0,033**
	Solos and depressed	150	14,25	4,95	2,157	140,951	
**Orientation**	Depressed	66	9,91	0,29	2,283	214	**0,003**
	Solos and depressed	150	9,71	0,67	3,001	213,976	
**Attention and calculation**	Depressed	66	4,82	0,46	1,78	214	**0,044**
	Solos and depressed	150	4,66	0,65	2,03	172,083	
**3 Words Memory**	Depressed	66	2,70	0,55	2,466	214	**0,007**
	Solos and depressed	150	2,45	0,74	2,752	162,794	
**Global cognitive function**	Depressed	66	29,14	0,99	3,248	214	**0**
	Solos and depressed	150	28,47	1,54	3,824	185,328	

Tabla 5c. Comparison of means between solos and “solos and depressed” groups.							
	Groups	Number	Median	standard deviation	t	Degrees of freedom	P-value
**Health status**	Solos	433	3,71	0,68	5,006	576	**0,000**
	Solos and depressed	145	3,37	0,78	4,667	221,242	
**Circulation problems (excluding varicose veins). intermittent claudication**	Solos	443	0,16	0,37	-2,585	591	**0,009**
	Solos and depressed	150	0,26	0,44	-2,378	225,112	
**Osteoarthritis**	Solos	444	0,33	0,47	-3,239	592	**<.001**
	Solos and depressed	150	0,48	0,50	-3,144	244,231	
**Arthritis or rheumatism**	Solos	444	0,15	0,36	-2,65	590	**0,008**
	Solos and depressed	148	0,24	0,43	-2,412	217,957	
**Asthma**	Solos	443	0,04	0,20	-3,167	590	**0,006**
	Solos and depressed	149	0,11	0,32	-2,556	189,814	
**Anxiety**	Solos	444	0,09	0,28	-9,886	592	**<.001**
	Solos and depressed	150	0,41	0,49	-7,654	183,197	
**Osteoporosis**	Solos	444	0,11	0,32	-3,025	592	**0,004**
	Solos and depressed	150	0,21	0,41	-2,674	212,935	
**Chronic back pain**	Solos	444	0,19	0,39	-4,11	592	**<.001**
	Solos and depressed	150	0,35	0,48	-3,731	220,782	
**Urinary tract problems (prostate. bladder)**	Solos	444	0,10	0,31	-2,092	591	**0,030**
	Solos and depressed	149	0,17	0,38	-1,89	217,491	
**During the last 4 weeks. to what extent has any type of pain made it difficult for you to do your usual work (paid work and domestic or care work) or your leisure activities?**	Solos	444	1,69	1,10	-3,272	592	**0,003**

	Solos and depressed	150	2,05	1,35	-2,959	219,651	
**Headache**	Solos	85	0,82	1,87	-2,501	130	**0,027**
	Solos and depressed	47	1,83	2,73	-2,252	70,435	
Feet pain	Solos	87	1,49	2,61	-0,122	132	0,903
	Solos and depressed	47	1,55	2,79	-0,119	89,033	
**Creatinine (mg/dL)**	Solos	425	0,86	0,23	2,51	560	**0,012**
	Solos and depressed	137	0,81	0,18	2,827	288,034	
**Monocyte percentage**	Solos	311	7,14	1,12	3,559	402	**0,000**
	Solos and depressed	93	6,67	1,17	3,467	145,533	
Age	Solos	444	64,64	10,09	0,079	592	0,937
	Solos and depressed	150	64,56	11,04	0,076	238,595	
**Level of satisfaction with your life in general**	Solos	344	7,04	1,17	2,963	472	**0,008**
	Solos and depressed	130	6,65	1,47	2,68	194,437	
**Level of satisfaction with your state of health**	Solos	364	6,66	1,43	5,508	493	**0,000**
	Solos and depressed	131	5,81	1,72	5,059	198,742	
**Level of satisfaction with the achievements you are currently achieving in life**	Solos	358	6,84	1,38	3,808	482	**0,000**
	Solos and depressed	126	6,27	1,57	3,58	197,102	
**Level of satisfaction with personal relationships**	Solos	305	7,32	0,98	2,496	407	**0,030**
	Solos and depressed	104	7,02	1,29	2,19	146,075	
**Level of satisfaction with how safe and secure you feel**	Solos	325	7,09	1,19	2,651	437	**0,020**
	Solos and depressed	114	6,72	1,54	2,347	162,875	
**Level of satisfaction with their feeling of belonging to a**	Solos	347	6,98	1,34	2,203	471	**0,045**

**community or group of people**							
	Solos and depressed	126	6,65	1,63	2,015	190,321	
**Number of diseases**	Solos	438	3,47	1,95	-8,381	581	**<.001**
	Solos and depressed	145	5,17	2,52	-7,376	203,906	
CO_task cancellation hits made	Solos	439	17,31	5,93	1,961	585	0,067
	Solos and depressed	148	16,17	6,74	1,841	228,475	
Semantic fluency	Solos	443	18,55	5,49	1,694	591	0,091
	Solos and depressed	150	17,63	6,35	1,576	228,927	
Phonological fluency	Solos	443	11,05	5,29	0,849	591	0,396
	Solos and depressed	150	10,61	5,96	0,801	233,518	
Number of objects identified	Solos	444	14,89	4,90	1,366	592	0,172
	Solos and depressed	150	14,25	4,95	1,359	254,474	
Orientation	Solos	444	9,82	0,48	2,2	592	0,062
	Solos and depressed	150	9,71	0,67	1,875	203,403	
**Attention and calculation**	Solos	444	4,78	0,55	2,14	592	**0,050**
	Solos and depressed	150	4,66	0,65	1,968	225,021	
3 Words Memory	Solos	444	2,37	0,77	-1,016	592	0,310
	Solos and depressed	150	2,45	0,74	-1,035	265,491	
Global cognitive function	Solos	444	28,62	1,47	1,103	592	0,270
	Solos and depressed	150	28,47	1,54	1,078	246,899	
