# Supplementary material for: Loneliness, Depression, and Genetics in the Elderly: Prognostic Factors of a Worse Health Condition?
Source: Int J Environ Res Public Health. 2022 Nov 22;19(23):15456. doi: 10.3390/ijerph192315456 (PMC9739711; doi:10.3390/ijerph192315456)
Supplement: Supplementary file 1 [file ijerph-19-15456-s001.zip › Supplementary Material S2. Tabla S2 Comparison of means between groups in men.pdf]

Tabla S2. Comparison of means between depressed individuals and solos groups in men.							
	Groups	Number	Median	standard deviation	t	Degrees of freedom	P-value
**heart attack**	Depressed	12	0,00	0,00	-1,01	253,00	**<.001**
	Solos	243	0,08	0,27	-4,53	242,00	
Angina pectoris	Depressed	12	0,00	0,00	-0,68	254,00	0,250
	Solos	244	0,04	0,19	-3,05	243,00	
**Circulation problems (excluding varicose veins). intermittent claudication**	Depressed	12	0,00	0,00	-1,25	253,00	**<.001**
	Solos	243	0,12	0,32	-5,61	242,00	
Anxiety	Depressed	12	0,25	0,45	3,09	254,00	0,073
	Solos	244	0,05	0,21	1,56	11,23	
Embolism / cerebral stroke	Depressed	12	0,00	0,00	-0,64	254,00	0,263
	Solos	244	0,03	0,18	-2,87	243,00	
**Malignant tumors/ Cancer**	Depressed	12	0,00	0,00	-1,22	253,00	**<.001**
	Solos	243	0,11	0,32	-5,50	242,00	
**Eosinophils (x1000/mm3)**	Depressed	12	2,51	19,08	2,14	238	**0,034**
	Solos	228	1,74	11,65	1,38	11,44	
Monocyte percentage	Depressed	8	76,38	100,70	0,90	165	0,369
	Solos	159	73,13	99,25	0,89	7,70	
Number of diseases	Depressed	12	3,92	2,23	1,42	252,00	0,078
	Solos	242	3,14	1,83	1,18	11,74	
**Orientation**	Depressed	12	10,00	0	1,16	254	**0,000**
	Solos	244	9,85	438	5,26	243,00	
**3 Words Memory**	Depressed	12	2,75	452	1,70	254	**0,014**
	Solos	244	2,36	786	2,78	14,50	
**How many hours a day do you spend sitting?**	Depressed	11	1,55	522	-2,56	253	**0,011**
	Solos	244	2,23	884	-4,11	12,74	
**Yale Sitting Index**	Depressed	11	1,55	522	-2,56	253	**0,011**
	Solos	244	2,23	884	-4,11	12,74	

Tabla 6b. Comparison of means between depressed individuals and “solos and depressed” groups in men.							
	Groups	Number	Median	standard deviation	t	Degrees of freedom	P-value
heart attack	Depressed	12	0,00	0,00	-0,78	50,00	0,220
	Solos and depressed	40	0,05	0,22	-1,43	39,00	
Angina pectoris	Depressed	12	0,00	0,00			
	Solos and depressed	40	0,00	0,00			
**Circulation problems (excluding varicose veins). intermittent claudication**	Depressed	12	0,00	0,00	-1,28	50,00	**0,012**
	Solos and depressed	40	0,13	0,34	-2,36	39,00	
Anxiety	Depressed	12	0,25	0,45	-0,17	50,00	0,434
	Solos and depressed	40	0,28	0,45	-0,17	18,13	
Embolism / cerebral stroke	Depressed	12	0,00	0,00			
	Solos and depressed	40	0,00	0,00			
**Malignant tumors/ Cancer**	Depressed	12	0,00	0,00	-1,13	50,00	**0,022**
	Solos and depressed	40	0,10	0,30	-2,08	39,00	
Eosinophils (x1000/mm3)	Depressed	12	2,51	19,08	1,19	45,00	0,242
	Solos and depressed	35	1,97	11,04	0,92	13,61	
Monocyte percentage	Depressed	8	76,38	100,70	1,87	27,00	0,073
	Solos and depressed	21	68,29	105,55	1,91	13,27	
Number of diseases	Depressed	12	3,92	2,23	-0,27	49,00	0,394
	Solos and depressed	39	4,10	2,02	-0,26	16,94	
**Orientation**	Depressed	12	10,00	0,00	1,21	50,00	**0,016**
	Solos and depressed	40	9,85	0,43	2,22	39,00	
3 Words Memory	Depressed	12	2,75	452,00	1,54	50,00	0,130
	Solos and depressed	40	2,40	744,00	1,99	30,46	
How many hours a day do you spend sitting?	Depressed	11	1,55	522,00	-1,81	48,00	0,077
	Solos and depressed	39	2,08	929,00	-2,45	29,60	
Yale Sitting Index	Depressed	11	1,55	522,00	-1,81	48,00	0,077
	Solos and depressed	39	2,08	929,00	-2,45	29,60	

Tabla 6c. Comparison of means between solos and “solos and depressed” groups in men.							
	Groups	Number	Median	standard deviation	t	Degrees of freedom	P-value
heart attack	Solos	243	0,08	0,27	0,63	281,00	0,265
	Solos and depressed	40	0,05	0,22	0,72	59,83	
**Angina pectoris**	Solos	244	0,04	0,19	1,23	282,00	**0,001**
	Solos and depressed	40	0,00	0,00	3,05	243,00	
Circulation problems (excluding varicose veins). intermittent claudication	Solos	243	0,12	0,32	-0,18	281,00	0,429
	Solos and depressed	40	0,13	0,34	-0,17	51,41	
**Anxiety**	Solos	244	0,05	0,21	-5,27	282,00	**0,001**
	Solos and depressed	40	0,28	0,45	-3,16	41,74	
**Embolism / cerebral stroke**	Solos	244	0,03	0,18	1,16	282,00	**0,002**
	Solos and depressed	40	0,00	0,00	2,87	243,00	
Malignant tumors/ Cancer	Solos	243	0,11	0,32	0,21	281,00	0,418
	Solos and depressed	40	0,10	0,30	0,21	53,74	
Eosinophils (x1000/mm3)	Solos	228	1,74	11,65	-1,10	0,26	0,271
	Solos and depressed	35	1,97	11,04	-1,15	46,43	
**Monocyte percentage**	Solos	159	73,13	99,25	2,09	0,18	**0,038**
	Solos and depressed	21	68,29	105,55	1,99	24,90	
**Number of diseases**	Solos	242	3,14	1,83	-3,01	0,28	**0,001**
	Solos and depressed	39	4,10	2,02	-2,79	48,49	
Orientation	Solos	244	9,85	0,44	0,03	0,28	0,974
	Solos and depressed	40	9,85	0,43	0,03	53,41	
3 Words Memory	Solos	244	2,36	0,79	-0,30	0,28	0,768
	Solos and depressed	40	2,40	0,74	-0,31	54,29	
How many hours a day do you spend sitting?	Solos	244	2,23	0,88	1,02	0,28	0,308
	Solos and depressed	39	2,08	0,93	0,99	49,63	
Yale Sitting Index	Solos	244	2,23	0,88	1,02	0,28	0,308
	Solos and depressed	39	2,08	0,93	0,99	49,63	
