# Supplementary material for: Loneliness, Depression, and Genetics in the Elderly: Prognostic Factors of a Worse Health Condition?
Source: Int J Environ Res Public Health. 2022 Nov 22;19(23):15456. doi: 10.3390/ijerph192315456 (PMC9739711; doi:10.3390/ijerph192315456)
Supplement: Supplementary file 1 [file ijerph-19-15456-s001.zip › Supplementary Material S3. Tabla S3. Comparison of means between groups in women.pdf]

Tabla S3. Comparison of means between depressed individuals and solos groups in women.							
	Groups	Number	Median	standard deviation	t	Degrees of freedom	P-value
Health status	Depressed	52	3,650	0,738	-0,142	244	0,887
	Solos	194	3,670	0,730	-0,141	79,827	
Heart attack	Depressed	54	0,060	0,231	3,416	252,000	0,083
	Solos	200	0,000	0,000	1,766	53,000	
**Anxiety**	Depressed	54	0,350	0,482	3,635	252,000	**0,004**
	Solos	200	0,140	0,348	3,024	68,591	
**Osteoporosis**	Depressed	54	0,110	0,317	-1,859	252,000	**0,032**
	Solos	200	0,230	0,419	-2,176	108,191	
Chronic back pain	Depressed	54	0,220	0,420	-0,420	252,000	0,675
	Solos	200	0,250	0,434	-0,428	86,135	
Urinary tract problems (prostate. bladder)	Depressed	54	0,060	0,231	0,164	252,000	0,870
	Solos	200	0,050	0,218	0,158	80,394	
During the last 4 weeks. to what extent has any type of pain made it difficult for you to do your usual work (paid work and domestic or care work) or your leisure activities?	Depressed	54	1,780	1,176	-0,924	252	0,356
	Solos	200	1,960	1,269	-0,966	89,213	
Headache	Depressed	11	1,270	2,533	1,253	65	0,215
	Solos	56	0,590	1,437	0,868	11,295	
**Feet pain**	Depressed	9	0,000	0,000	-1,987	65	**0,000**
	Solos	58	1,760	2,638	-5,077	57,000	
Eosinophils (x1000/mm3)	Depressed	51	0,159	0,126	-0,726	243	0,469
	Solos	194	0,177	0,161	-0,837	97,674	
Monocyte percentage	Depressed	37	6,849	1,223	-0,539	187	0,591

	Solos	152	6,968	1,210	-<br>0,535	54,470	
Level of satisfaction with your life in general	Depressed	37	7,050	1,224	-<br>0,108	196	0,914
	Solos	161	7,070	0,997	-<br>0,095	47,581	
Level of satisfaction with his health	Depressed	44	6,300	1,250	-<br>1,228	210	0,221
	Solos	168	6,580	1,382	-<br>1,302	73,029	
Level of satisfaction with the achievements you are currently achieving in life	Depressed	36	7,170	1,134	1,338	193	0,183
	Solos	159	6,850	1,318	1,471	58,469	
Level of satisfaction with personal relationships	Depressed	22	7,590	0,590	1,108	159	0,270
	Solos	139	7,380	0,855	1,443	36,630	
Level of satisfaction with how safe and secure you feel	Depressed	28	7,360	0,780	0,426	173	0,671
	Solos	147	7,270	1,087	0,532	49,438	
Level of satisfaction with their feeling of belonging to a community or group of people	Depressed	32	7,250	0,950	0,978	189	0,329
	Solos	159	7,020	1,265	1,181	55,671	
Number of diseases	Depressed	53	4,434	2,275	1,699	0,247	0,091
	Solos	196	3,888	2,020	1,587	75,624	
Orientation	Depressed	54	9,890	0,317	1,316	252	0,189
	Solos	200	9,790	0,527	1,735	140,489	
Attention and calculation	Depressed	54	4,800	0,491	1,328	252	0,186
	Solos	200	4,670	0,651	1,558	108,730	
**3 Words Memory**	Depressed	54	2,690	0,577	2,709	252	**0,002**
	Solos	200	2,390	0,742	3,126	105,304	
**Reading**	Depressed	54	1,000	0,000	1,046	252	**0,045**
	Solos	200	0,980	0,140	2,015	199,000	
**Global cognitive function**	Depressed	54	29,060	1,017	2,714	252	**0,001**
	Solos	200	28,440	1,593	3,477	131,152	
**How many minutes of nap do you usually sleep each day?**	Depressed	54	20,390	25,574	2,074	252	**0,039**
	Solos	200	13,060	22,325	1,918	76,195	
How often do you wake up rested in the morning?	Depressed	54	2,350	0,850	-<br>1,578	250	0,116

	Solos	198	2,530	0,703	- 1,416	73,905	
Sleeping difficulties	Depressed	54	1,930	1,330	- 0,403	251	0,687
	Solos	199	2,000	1,159	- 0,373	76,242	
Waking up multiple times while sleeping	Depressed	54	2,690	1,464	1,276	251	0,203
	Solos	199	2,430	1,277	1,179	76,248	
How many hours a day do you spend moving while doing things?	Depressed	54	3,590	1,108	- 1,724	251	0,086
	Solos	199	3,870	1,054	- 1,675	80,918	
How many hours a day do you spend on your feet?	Depressed	54	3,090	1,233	- 1,708	251	0,089
	Solos	199	3,420	1,240	- 1,713	84,391	
Yale Movement Index	Depressed	54	10,780	3,323	- 1,724	251	0,086
	Solos	199	11,620	3,161	- 1,675	80,918	
Yale Standing Index	Depressed	54	6,190	2,465	- 1,708	251	0,089
	Solos	199	6,830	2,480	- 1,713	84,391	
CO_task cancellation row has crossed out the last letter	Depressed	54	11,500	4,325	0,445	250	0,657
	Solos	198	11,190	4,650	0,464	89,297	
CO_task cancellation last letter crossed out	Depressed	54	######	130,259	0,399	250	0,690
	Solos	198	######	140,123	0,416	89,338	
CO_task cancellation hits made	Depressed	54	18,520	5,524	1,430	250	0,154
	Solos	198	17,230	5,974	1,496	89,742	
CO_task cancellation commission errors made	Depressed	54	0,300	1,253	2,195	250	0,206
	Solos	198	0,080	0,348	1,280	55,246	
CO_task cancellation hits should be	Depressed	54	26,910	11,042	0,424	250	0,672
	Solos	198	26,140	11,966	0,444	89,903	
Semantic fluency	Depressed	54	19,850	5,343	1,936	252	0,054
	Solos	200	18,250	5,431	1,954	84,936	
**Phonological fluency**	Depressed	54	12,540	3,830	1,848	252	**0,026**
	Solos	200	11,080	5,457	2,255	117,614	

**Number of objects identified**	Depressed	54	15,570	4,407	2,133	252	**0,034**
	Solos	200	13,980	4,991	2,291	93,103	

Tabla 7b. Comparison of means between depressed individuals and "solos and depressed" groups in women.							
	Groups	Number	Median	standard deviation	t	Degrees of freedom	P-value
**Health status**	Depressed	52	3,650	0,738	2,666	158,00	**0,008**
	Solos and depressed	108	3,310	0,791	2,731	107,36	
Heart attack	Depressed	54	0,060	0,231	0,568	162,00	0,571
	Solos and depressed	110	0,040	0,188	0,530	88,47	
Anxiety	Depressed	54	0,350	0,482	-1,360	162,00	0,171
	Solos and depressed	110	0,460	0,501	-1,378	109,18	
**Osteoporosis**	Depressed	54	0,110	0,317	-2,489	162,00	**0,006**
	Solos and depressed	110	0,280	0,452	-2,799	142,47	
**Chronic back pain**	Depressed	54	0,220	0,420	-2,278	162,00	**0,018**
	Solos and depressed	110	0,400	0,492	-2,405	121,74	
Urinary tract problems (prostate. bladder)	Depressed	54	0,060	0,231	-1,695	162,00	0,053
	Solos and depressed	110	0,150	0,354	-1,948	149,18	
**During the last 4 weeks. to what extent has any type of pain made it difficult for you to do your usual work (paid work and domestic or care work) or your leisure activities?**	Depressed	54	1,780	1,176	-2,122	162,00	**0,025**
	Solos and depressed	110	2,250	1,430	-2,267	125,64	
Headache	Depressed	11	1,270	2,533	-0,628	50,00	0,533
	Solos and depressed	41	1,850	2,771	-0,662	17,01	

**Feet pain**	Depressed	9	0,000	0,000	-1,711	48,00	**0,001**
	Solos and depressed	41	1,590	2,757	-3,683	40,00	
Eosinophils (x1000/mm3)	Depressed	51	0,159	0,126	1,163	147,00	0,247
	Solos and depressed	98	0,137	0,100	1,083	83,68	
Monocyte percentage	Depressed	37	6,849	1,223	0,918	107,00	0,361
	Solos and depressed	72	6,624	1,206	0,914	71,87	
Level of satisfaction with your life in general	Depressed	37	7,050	1,224	1,703	131,00	0,091
	Solos and depressed	96	6,570	1,541	1,885	81,86	
**Level of satisfaction with his health**	Depressed	44	6,300	1,250	2,525	138,00	**0,005**
	Solos and depressed	96	5,560	1,728	2,840	112,33	
**Level of satisfaction with the achievements you are currently achieving in life**	Depressed	36	7,170	1,134	3,227	127,00	**0,000**
	Solos and depressed	93	6,220	1,621	3,763	90,68	
**Level of satisfaction with personal relationships**	Depressed	22	7,590	0,590	2,122	97,00	**0,002**
	Solos and depressed	77	6,960	1,352	3,166	80,89	
**Level of satisfaction with how safe and secure you feel**	Depressed	28	7,360	0,780	2,416	107,00	**0,001**
	Solos and depressed	81	6,580	1,635	3,321	96,29	
**Level of satisfaction with their feeling of belonging to a community or group of people**	Depressed	32	7,250	0,950	2,148	120,00	**0,006**
	Solos and depressed	90	6,560	1,736	2,795	99,44	
**Number of diseases**	Depressed	53	4,434	2,275	-2,686	157,00	**0,008**

	Solos and depressed	106	5,557	2,582	-2,802	116,63	
**Orientation**	Depressed	54	9,890	0,317	2,159	162,00	**0,007**
	Solos and depressed	110	9,660	0,733	2,742	160,11	
**Attention and calculation**	Depressed	54	4,800	0,491	1,863	162,00	**0,038**
	Solos and depressed	110	4,600	0,693	2,089	141,65	
**3 Words Memory**	Depressed	54	2,690	0,577	1,934	162,00	**0,038**
	Solos and depressed	110	2,460	0,738	2,101	131,15	
Reading	Depressed	54	1,000	0,000	0,700	162,00	0,485
	Solos and depressed	110	0,990	0,095	1,000	109,00	
**Global cognitive function**	Depressed	54	#####	1,017	2,865	162,00	**0,001**
	Solos and depressed	110	#####	1,624	3,332	152,51	
How many minutes of nap do you usually sleep each day?	Depressed	54	#####	25,574	0,716	162,00	0,475
	Solos and depressed	110	#####	26,804	0,728	110,02	
How often do you wake up rested in the morning?	Depressed	54	2,350	0,850	1,080	162,00	0,282
	Solos and depressed	110	2,200	0,844	1,078	104,73	
**Sleeping difficulties**	Depressed	54	1,930	1,330	-2,094	162,00	**0,038**
	Solos and depressed	110	2,410	1,416	-2,140	111,63	
Waking up multiple times while sleeping	Depressed	54	2,690	1,464	-0,407	162,00	0,684
	Solos and depressed	110	2,780	1,410	-0,402	101,97	
How many hours a day do you spend moving while doing things?	Depressed	54	3,590	1,108	0,641	161,00	0,523
	Solos and depressed	109	3,470	1,199	0,658	113,56	
How many hours a day do you spend on your feet?	Depressed	54	3,090	1,233	0,844	162,00	0,400

	Solos and depressed	110	2,910	1,345	0,869	114,08	
Yale Movement Index	Depressed	54	#####	3,323	0,641	161,00	0,523
	Solos and depressed	109	#####	3,596	0,658	113,56	
Yale Standing Index	Depressed	54	6,190	2,465	0,844	162,00	0,400
	Solos and depressed	110	5,820	2,689	0,869	114,08	
**CO_task cancellation row has crossed out the last letter**	Depressed	54	#####	4,325	1,697	160,00	**0,046**
	Solos and depressed	108	#####	4,704	1,746	114,40	
**CO_task cancellation last letter crossed out**	Depressed	54	#####	130,259	1,653	160,00	**0,050**
	Solos and depressed	108	#####	141,471	1,699	114,27	
**CO_task cancellation hits made**	Depressed	54	#####	5,524	2,931	160,00	**0,002**
	Solos and depressed	108	#####	6,369	3,074	120,50	
CO_task cancellation commission errors made	Depressed	54	0,300	1,253	-0,787	160,00	0,216
	Solos and depressed	108	0,510	1,780	-0,881	142,17	
**CO_task cancellation hits should be**	Depressed	54	#####	11,042	1,862	160,00	**0,032**
	Solos and depressed	108	#####	11,614	1,894	111,02	
**Semantic fluency**	Depressed	54	#####	5,343	2,299	162,00	**0,011**
	Solos and depressed	110	#####	6,212	2,421	120,83	
**Phonological fluency**	Depressed	54	#####	3,830	2,362	162,00	**0,004**
	Solos and depressed	110	#####	5,893	2,717	149,56	
**Number of objects identified**	Depressed	54	#####	4,407	2,490	162,00	**0,007**
	Solos and depressed	110	#####	5,033	2,606	118,90	

Tabla 7c. Comparison of means between solos and “solos and depressed” groups in women.

	Groups	Number	Median	standard deviation	t	Degrees of freedom	P-value
**Health status**	Solos	194	3,670	0,730	4,036	300,000	**0,000**
	Solos and depressed	108	3,310	0,791	3,945	206,826	
**Heart attack**	Solos	200	0,000	0,000	-2,738	308,000	**0,045**
	Solos and depressed	110	0,040	0,188	-2,028	109,000	
**Anxiety**	Solos	200	0,140	0,348	-6,672	308,000	**0,000**
	Solos and depressed	110	0,460	0,501	-6,024	168,006	
Osteoporosis	Solos	200	0,230	0,419	-1,111	308,000	0,278
	Solos and depressed	110	0,280	0,452	-1,087	210,475	
**Chronic back pain**	Solos	200	0,250	0,434	-2,774	308,000	**0,008**
	Solos and depressed	110	0,400	0,492	-2,675	201,987	
**Urinary tract problems (prostate. bladder)**	Solos	200	0,050	0,218	-2,932	308,000	**0,011**
	Solos and depressed	110	0,150	0,354	-2,570	155,671	
During the last 4 weeks. to what extent has any type of pain made it difficult for you to do your usual work (paid work and domestic or care work) or your leisure activities?	Solos	200	1,960	1,269	-1,900	308,000	0,058
	Solos and depressed	110	2,250	1,430	-1,835	203,053	
**Headache**	Solos	56	0,590	1,437	-2,923	95,000	**0,010**
	Solos and depressed	41	1,850	2,771	-2,671	55,729	
Feet pain	Solos	58	1,760	2,638	0,316	97,000	0,753
	Solos and depressed	41	1,590	2,757	0,314	83,881	
**Eosinophils (x1000/mm3)**	Solos	194	0,177	0,161	2,227	290,000	**0,027**

	Solos and depressed	98	0,137	0,100	2,580	277,826	
**Monocyte percentage**	Solos	152	6,968	1,210	1,994	222,000	**0,047**
	Solos and depressed	72	6,624	1,206	1,996	139,915	
**Level of satisfaction with your life in general**	Solos	161	7,070	0,997	3,168	255,000	**0,005**
	Solos and depressed	96	6,570	1,541	2,854	143,090	
**Level of satisfaction with his health**	Solos	168	6,580	1,382	5,230	262,000	**0,000**
	Solos and depressed	96	5,560	1,728	4,925	164,643	
**Level of satisfaction with the achievements you are currently achieving in life**	Solos	159	6,850	1,318	3,380	250,000	**0,002**
	Solos and depressed	93	6,220	1,621	3,203	162,728	
**Level of satisfaction with personal relationships**	Solos	139	7,380	0,855	2,795	214,000	**0,015**
	Solos and depressed	77	6,960	1,352	2,468	110,398	
**Level of satisfaction with how safe and secure you feel**	Solos	147	7,270	1,087	3,786	226,000	**0,001**
	Solos and depressed	81	6,580	1,635	3,382	119,876	
**Level of satisfaction with their feeling of belonging to a community or group of people**	Solos	159	7,020	1,265	2,418	247,000	**0,028**
	Solos and depressed	90	6,560	1,736	2,220	143,271	
**Number of diseases**	Solos	196	3,888	2,020	-6,199	300,000	**0,000**

	Solos and depressed	106	5,557	2,582	-5,768	175,626	
Orientation	Solos	200	9,790	0,527	1,752	308,000	0,081
	Solos and depressed	110	9,660	0,733	1,596	172,078	
Attention and calculation	Solos	200	4,670	0,651	0,885	308,000	0,377
	Solos and depressed	110	4,600	0,693	0,869	212,784	
3 Words Memory	Solos	200	2,390	0,742	-0,838	308,000	0,403
	Solos and depressed	110	2,460	0,738	-0,839	225,720	
Reading	Solos	200	0,980	0,140	-0,728	308,000	0,467
	Solos and depressed	110	0,990	0,095	-0,811	294,505	
Global cognitive function	Solos	200	28,440	1,593	0,375	308,000	0,708
	Solos and depressed	110	28,360	1,624	0,373	221,029	
How many minutes of nap do you usually sleep each day?	Solos	200	13,060	22,325	-1,469	308,000	0,143
	Solos and depressed	110	17,250	26,804	-1,393	192,677	
**How often do you wake up rested in the morning?**	Solos	198	2,530	0,703	3,674	306,000	**0,001**
	Solos and depressed	110	2,200	0,844	3,488	193,339	
**Sleeping difficulties**	Solos	199	2,000	1,159	-2,741	307,000	**0,010**
	Solos and depressed	110	2,410	1,416	-2,588	190,306	
**Waking up multiple times while sleeping**	Solos	199	2,430	1,277	-2,252	307,000	**0,030**
	Solos and depressed	110	2,780	1,410	-2,188	206,765	
**How many hours a day do you spend moving while doing things?**	Solos	199	3,870	1,054	3,081	306,000	**0,003**

	Solos and depressed	109	3,470	1,199	2,968	199,327	
**How many hours a day do you spend on your feet?**	Solos	199	3,420	1,240	3,345	307,000	**0,001**
	Solos and depressed	110	2,910	1,345	3,268	209,992	
**Yale Movement Index**	Solos	199	11,620	3,161	3,081	306,000	**0,003**
	Solos and depressed	109	10,400	3,596	2,968	199,327	
**Yale Standing Index**	Solos	199	6,830	2,480	3,345	307,000	**0,001**
	Solos and depressed	110	5,820	2,689	3,268	209,992	
**CO_task cancellation row has crossed out the last letter**	Solos	198	11,190	4,650	1,760	304,000	**0,040**
	Solos and depressed	108	10,200	4,704	1,754	217,861	
**CO_task cancellation last letter crossed out**	Solos	198	322,450	140,123	1,755	304,000	**0,040**
	Solos and depressed	108	292,930	141,471	1,750	218,214	
**CO_task cancellation hits made**	Solos	198	17,230	5,974	2,310	304,000	**0,011**
	Solos and depressed	108	15,540	6,369	2,267	208,277	
**CO_task cancellation commission errors made**	Solos	198	0,080	0,348	-3,317	304,000	**0,007**
	Solos and depressed	108	0,510	1,780	-2,505	111,483	
**CO_task cancellation hits should be**	Solos	198	26,140	11,966	1,962	304,000	**0,025**
	Solos and depressed	108	23,360	11,614	1,980	225,687	
Semantic fluency	Solos	200	18,250	5,431	0,977	308,000	0,165
	Solos and depressed	110	17,580	6,212	0,939	200,481	

Phonological fluency	Solos	200	11,080	5,457	0,931	308,000	0,176
	Solos and depressed	110	10,450	5,893	0,910	210,421	
Number of objects identified	Solos	200	13,980	4,991	0,685	308,000	0,247
	Solos and depressed	110	13,570	5,033	0,684	223,075	
