# Supplementary material for: Loneliness, Depression, and Genetics in the Elderly: Prognostic Factors of a Worse Health Condition?
Source: Int J Environ Res Public Health. 2022 Nov 22;19(23):15456. doi: 10.3390/ijerph192315456 (PMC9739711; doi:10.3390/ijerph192315456)
Supplement: Supplementary file 1 [file ijerph-19-15456-s001.zip › Supplementary Material S4. Table S4. Logistic regression analysis SNPs and groups.pdf]

Table S4. Logistic regression analysis between SNPs and controls and "solo and depressed" groups.						
SNP	Model	Genotype	**CONTROL** (N)	**SOLO AND DEPRESSED** (N)	OR (95% CI)	P-value
rs182549	Log-additive	CC<br><br>CT<br>TT	38<br><br>59<br>23	14<br><br>16<br>1	0,51 (0,27-0,96)	0,03
rs2154110	Recessive	TT/GT<br>GG	119<br>1	27<br>4	1<br>17,63 (1,89-164,09)	 0,0035
rs4902100	Recessive	AA/AG<br>GG	119<br>1	27<br>4	1<br>17,63 (1,89-164,09)	 0,0035
rs6265	Log-additive	CC<br><br>CT<br>TT	81<br><br>39<br>0	14<br><br>14<br>3	2,94 (1,42-6,11)	0,0034

Table 8b. Logistic regression analysis between SNPs and controls and depressed groups.						
SNP	Model	Genotype	**CONTROLS** (N)	**DEPRESSED** (N)	OR (95% CI)	P-value
rs10904887	Dominant	TT<br>CT/CC	33<br>87	4<br>1	1<br>0.09 (0.01-0.88)	0.017
rs10904896	Dominant	GG<br>AG/AA	33<br>87	4<br>1	1<br>0.09(0.01-0.88)	 0.017
rs12335203	Log-additive	TT<br>CT<br>CC	25<br>67<br>28	4<br>1<br>0	0.09(0.01-0.74)	 <br>0,0041
rs2154110	Log-additive	TT<br>GT<br>GG	64<br>55<br>1	1<br>3<br>1	6.88 (1.03-46.13)	0.031
rs391300	Log-additive	CC<br>CT<br>TT<br>GG	27<br>66<br>27<br>1	4<br>1<br>0<br>1	0.09(0.01-0.80)	0.0056

Table 8C. Logistic regression analysis between SNPs and controls and solo groups.						
SNP	Model	Genotype	**CONTROLS** (N)	**SOLO** (N)	OR (95% CI)	P-value

rs10509637	Dominant	AA<br>AG/GG	79<br>41	118<br>35	1<br>0.57 (0.33-0.99)	0.039
rs12335203	Dominant	CC<br>CT/TT	28<br>92	53<br>100	1<br>0.57(0.34-0.98)	0.041
rs2154110	Codominant	TT<br>GT<br>GG	64<br>55<br>1	91<br>48<br>14	1<br><br>9.85(1.26-76.78)	0.0007
rs391300	Dominant	CC<br>CT/TT	27<br>93	60<br>92	1<br>0.45(0.26-0.76)	0.0026
rs4902100	Codominant	AA<br>AG<br>GG	67<br>52<br>1	92<br>47<br>14	1<br><br>10.20(1.31-79.44)	0.001
rs7675998	Dominant	GG<br>AG/AA	56<br>64	95<br>58	1<br>0.53(0.33-0.87)	0.011
rs7412	Log-additive	CC<br>TC<br>TT	98<br>19<br>3	138<br>14<br>1	0.51(0.27-0.96)	0.032
